# River Plume Liftoff Dynamics and Surface Expressions

**DOI:** 10.1029/2019WR026475

**Published:** 2020-07-16

**Authors:** R. A. Branch, A. R. Horner‐Devine, N. Kumar, A. R. Poggioli

**Affiliations:** ^1^ Department of Civil and Environmental Engineering University of Washington Seattle WA USA; ^2^ Now at Pacific Northwest National Laboratory Seattle WA USA; ^3^ Materials Science Division Lawrence Berkeley National Laboratory Berkeley CA USA

**Keywords:** river discharge, ROMS, ocean modeling, SWOT

## Abstract

The water surface expression of liftoff and its dependence on discharge are examined using numerical simulations with the Regional Ocean Modeling System (ROMS). Liftoff is the process by which buoyant river water separates from the bed and flows over denser saltwater. During low‐discharge conditions liftoff occurs in the river and is accompanied by a change in the surface slope. During high‐discharge conditions liftoff occurs outside the mouth and generates a ridge on the water surface. The location and height of the ridge can be described by analytical equations in terms of discharge, shelf slope, and river mouth aspect ratio. The offshore distance and height of the ridge are proportional to the river discharge and vary inversely with river mouth aspect ratio. For steep shelf slopes liftoff occurs close to the river mouth and generates a large ridge. The ridge is modified, but not eliminated, by the presence of tides. The water surface slope change at the ridge peak is large enough to be detected by the upcoming Surface Water and Ocean Topography (SWOT) altimeter and can be used to identify the liftoff location during high discharge. However, during low discharge the water surface slope change at the liftoff location is too small to be detected by SWOT. These results indicate that remote measurements of the presence or absence of the ridge may be useful to distinguish between low and high flows, and remote measurements of the ridge location or height could be used to estimate freshwater discharge.

## Key Points

- River discharge generates a ridge on the water surface at the liftoff location that may be remotely detectable
- Liftoff location and ridge height are predicted by equations in terms of discharge, shelf slope, and river mouth aspect ratio
- Remote measurements of the location or height of the ridge could be used to estimate discharge

## 1 Introduction

Coastal river discharge carries nutrients, sediments, and contaminants into the coastal ocean, where the river water and its constituents play a central role in the circulation, morphology, and ecosystem function of coastal waters (Barkan et al., 2017; Hickey & Banas, 2003; Hickey et al., 2010; Syvitski et al., 2003). Accurate, distributed measurements of coastal river discharge are needed in order to understand river influences on coastal waters globally.

Discharge is traditionally measured using in situ river stage gauges and rating curves (Baldassarre & Montanari, 2009), but measurements are sparse in some parts of the world due to either inaccessibility or economic constraints. Recently, researchers have been evaluating the feasibility of using remote measurements (Bjerklie et al., 2003; Durand et al., 2016; Nickles et al., 2019; Tuozzolo et al., 2019), which offer global coverage and the ability to estimate discharge in locations where it is difficult to install in situ sensors. LeFavour and Alsdorf (2005) and Altenau et al. (2019) use remotely sensed water surface slope data to estimate discharge based on Manning's equation. Manning's equation is an empirical formula for the average velocity of uniform flow due to the balance between friction and gravity in an open channel (Manning et al., 1890). It can be converted to an equation for discharge, *Q*, using *Q*=*AV*, which yields $Q=\frac{1}{n}AR^{2/3}\sqrt{S}$, where *A* is the cross‐sectional area, *R* is the hydraulic radius, *S* is the river surface slope, and *n* is Manning's roughness coefficient. If the surface slope is measured by a satellite instrument and *A*, *R*, and *n* are estimated, then *Q* can be calculated. The requirement of uniform flow limits the applicability of this approach to river reaches where the water depth is constant and the surface slope is parallel to the bed. Uniform flow conditions generally only occur well upstream of the river mouth and not in the 10–100 km adjustment region upriver of the mouth (Chatanantavet et al., 2012; Lamb et al., 2012). Between the coast and the region of uniform flow the hydraulic regime is in either a state of drawdown (M2 profile) or backwater (M1 profile), resulting in convex or concave water surface profiles, respectively (Sturm, 2010). Thus, estimates of discharge using Manning's equation and the water surface slope here will be overestimated/underestimated. Alternate approaches not limited to uniform flow regions are necessary if remote sensing data are to be used to estimate river discharge near the coast. Here we investigate the dynamics of the river plume liftoff and evaluate whether remote measurements of its surface expression could be exploited to give a more accurate estimate of the amount of freshwater entering the coastal waters.

## 2 Background

Liftoff occurs when buoyant freshwater from the river detaches from the bottom and flows over dense salty ocean water. The liftoff process is governed by two‐layer hydraulics (Armi & Farmer, 1986) and described using the upper layer Froude number, which is the ratio of the depth‐averaged freshwater velocity, ${ū}_{1}$, to the gravity current propagation speed

(1) $$Fr_{1}=\frac{{ū}_{1}}{\sqrt{g^{\prime }h_{1}}},$$

where *g* *′* is the reduced gravitational acceleration (Δ*ρ*_0_/*ρ*_ocean_)*g*, Δ*ρ*_0_ is the density contrast between fresh and ocean water, and *h*_1_ is the depth of the freshwater flow (Geyer & Ralston, 2011). Liftoff occurs when the flow speed is reduced to the gravity current speed, that is, *Fr*_1_=1 (MacDonald & Geyer, 2004, 2005). This transition, and thus the liftoff location, occurs in the river channel during low‐discharge conditions or outside the river mouth during high‐discharge conditions.

**Figure 1:**
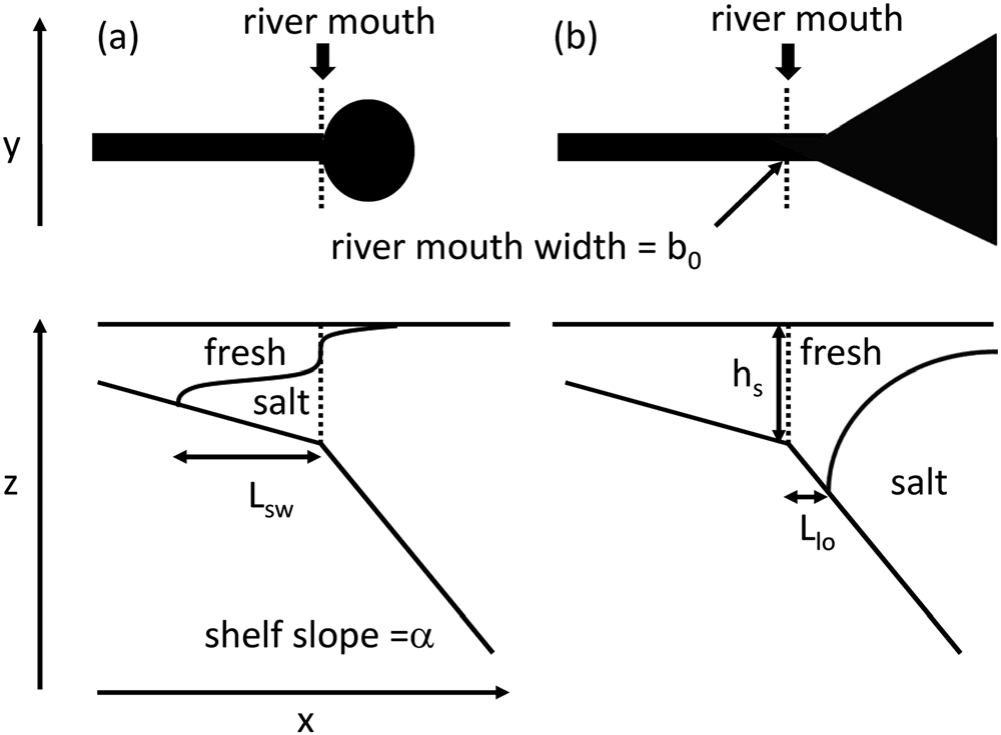
Schematic for (a) low discharge: (*F* *r*_*f*_<1) small plume with the salt wedge propagating up the river. The upper panel shows the shape of the surface freshwater in black. (b) High discharge: (*F* *r*_*f*_>1) a seaward directed jet at the mouth forming a large plume. The freshwater stays attached to the bottom of the shelf until liftoff. The width of the river mouth is *b*_0_, the shelf slope is *α*, the depth of the water at the mouth is *h*_*s*_, the length of the salt wedge is *L*_sw_, and the liftoff length is *L*_lo_.

Typically, discharge near the river mouth is described in terms of the freshwater Froude number

(2) $$Fr_{f}=\frac{ū}{\sqrt{g^{\prime }h_{s}}},$$

where ū and *h*_*s*_ are the depth‐averaged velocity and shoreline depth, respectively. The shoreline depth is defined as the total water depth at the river mouth. The freshwater Froude number represents the ratio of the depth‐averaged velocity to the gravity current propagation speed ( $\sqrt{g^{\prime }h_{s}}$) at the river mouth. During low‐discharge conditions, the gravity current speed exceeds the depth‐averaged velocity, *Fr*_*f*_<1 and the salt wedge propagates up into the channel a distance *L*_sw_. Under these conditions, the liftoff location is defined as the location of the toe of the salt wedge (Figure 1a). During high‐discharge conditions, the depth averaged velocity exceeds the gravity current speed, *Fr*_*f*_>1 and the salt water is forced out of the river channel. Under these conditions the freshwater stays attached to the shelf floor for a distance *L*_lo_ offshore until liftoff (Figure 1b). This distance, *L*_lo_, is called the liftoff length.

For a rectangular river, *Fr*_*f*_ can be expressed in terms of discharge, *Q*, as

(3) $$Fr_{f}=\frac{Q}{b_{0}\sqrt{g^{\prime }h_{s}^{3}}},$$

where *b*_0_ is the width of the river at the mouth. Liftoff occurs at the river mouth when *Fr*_1_=*Fr*_*f*_=1.

Most rivers are usually in a state of low discharge (*Fr*_*f*_<1); however, understanding the dynamics during high discharge events is important because they are the primary drivers of morphological change (Lamb et al., 2012). The process of offshore liftoff has been studied for decades due to the importance of buoyant surface jets from river and power plant outflows (Jones et al., 2007; MacDonald & Geyer, 2005; Safaie, 1979). High discharge flow enters the ocean as a jet, slows due to lateral spreading and increased depth, and then lifts off when the upper layer Froude number reaches one. Using an analytical model, Poggioli and Horner‐Devine (2018) predict that a ridge forms on the water surface with its peak at the liftoff location. The ridge becomes taller and moves further offshore as discharge increases. To our knowledge this ridge has not been measured yet, although the process of offshore liftoff has been studied before.

**Figure 2:**
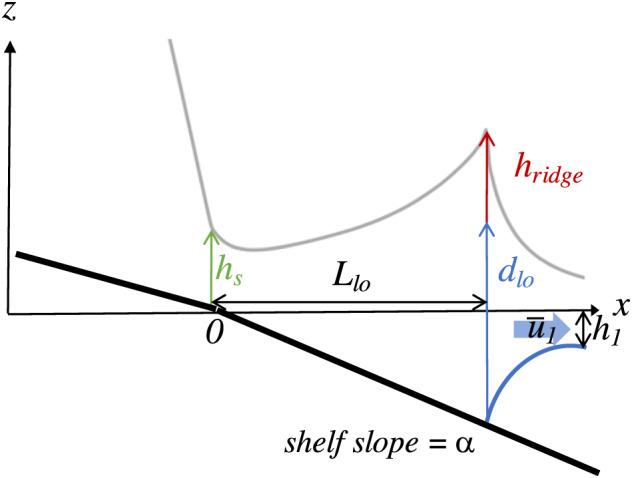
Side view schematic during high discharge showing ridge height variables. The river mouth is located at *x*=0 and river flow is from left to right. The scale of the water surface profile (gray line) is exaggerated to emphasize the shape of the offshore ridge. The thick black line and the blue curved line are the river/shelf bottom and the lower interface of the plume, respectively. The following parameters are labeled and described in the text: *h*_*s*_ (shoreline depth), *L*_lo_ (liftoff length), *h*_ridge_ (ridge height), *d*_lo_ (depth at liftoff), *u*_1_ (velocity in the plume layer), and *h*_1_ (thickness of the plume layer).

Previous studies of offshore liftoff have led to equations for either the depth at liftoff or the liftoff length. Safaie (1979) used laboratory data to obtain an empirical equation for the depth at liftoff, $d_{\text{lo}}=0.914Fr_{f}^{1/2}h_{s}$. This equation can be converted to an equation for the liftoff length by approximating the depth at liftoff as *h*_*s*_+*αL*_lo_, where *α* is the shelf slope. A schematic description of the liftoff process and the system parameters is shown in Figure 2. Defining the river mouth aspect ratio as *R*_*A*_=*b*_0_/*h*_*s*_, the nondimensionalized liftoff length predicted by the Safaie (1979) experiments is

(4) $$\frac{L_{\text{lo}}}{b_{0}}=\frac{1}{\alpha R_{A}}(0.914Fr_{f}^{1/2}-1).$$

Jones et al. (2007) used scaling analysis based on the momentum and buoyancy of the discharge to derive a jet‐to‐plume length scale, $L_{M}=ū{\left(b_{0}h_{s}\right)}^{1/4}/\sqrt{g^{\prime }}$, that can be expressed in terms of the freshwater Froude number as

(5) $$\frac{L_{\text{lo}}}{b_{0}}={\left(\frac{1}{R_{A}}\right)}^{3/4}Fr_{f}.$$

This equation was tested with measurements at the Columbia River by Kilcher and Nash (2010) who found their observations to be consistent with Equation 5, but also emphasized that liftoff will only occur when the water depth is greater than one third of the jet‐to‐plume length scale. Therefore, any equation for the liftoff length should incorporate the depth dependence resulting from the shelf slope. Using the condition that liftoff occurs when the upper layer Froude number equals one, Geyer and Ralston (2011) showed that liftoff in the estuary channel occurs when the depth is

(6) $$d_{\text{lo}}={\left(\frac{Q^{2}}{B^{2}g^{\prime }}\right)}^{1/3}$$

where *B* is the width of the estuary. If we assume that the width of the freshwater jet offshore is the same as the width of the estuary (*b*_0_=*B*) and that the depth at liftoff is *h*_*s*_+*αL*_lo_, then this equation predicts the following liftoff length outside the river mouth as

(7) $$\frac{L_{\text{lo}}}{b_{0}}=\frac{1}{R_{A}\alpha }(Fr_{f}^{2/3}-1).$$

The assumption that the width of the freshwater jet offshore is *b*_0_ implies that the plume does not spread outside of the river mouth. While this assumption may hold for some cases, plume spreading may be important in determining the liftoff length as will be demonstrated in this work.

Recently, Poggioli and Horner‐Devine (2018) used a two‐layer hydraulic model of the river, estuary, and near‐field river plume to study liftoff and included lateral plume spreading due to buoyancy. The model is hydrostatic, with the density and velocity assumed to be uniform in each layer and the velocity in the lower layer assumed to be negligible. A fit of the two‐layer hydraulic model gave an expression for the nondimensionalized liftoff length as

(8) $$\frac{L_{\text{lo}}}{b_{0}}=\gamma {\left(Fr_{f}-1\right)}^{n}$$

where *γ* and *n* are dimensionless geometric constants that are assumed to vary with *b*_0_, *h*_*s*_, and *α*. Increasing the shelf slope was found to decrease *γ* which was found to be in the range of *O*(10^−2^−1). A reasonable range of *n* was 1<*n*<1.4 and it was found to be only weakly dependent upon *b*_0_, *h*_*s*_, and *α*. As *γ* and *n* are not known a priori, Equation 8 describes the relationship between the liftoff length and the discharge but cannot be solved for the liftoff length of a specific river system even if *Q*, *b*_0_, *h*_*s*_, and *α* are known. The full model can be run to solve for the liftoff length based on specific values of *Q*, *b*_0_, *h*_*s*_, and *α*. A single equation that relates either liftoff length or ridge height to discharge in terms of *b*_0_, *h*_*s*_, and *α* would be useful for quantifying discharge if a remote measurement of the liftoff length or ridge height is possible. In order to test equations for the liftoff length and ridge height, we simulate river discharge into the coastal ocean using a three‐dimensional numerical model that captures more of the physical processes than the two‐layer hydraulic model.

Numerical models are often used to study river plume dynamics. Idealized numerical models with simplified river and coastal bathymetry were extensively utilized to study coastal ocean dynamics driven by buoyancy inputs (Garvine, 1982, 1984, 1987; O'Donnell, 1990). More recently, three‐dimensional numerical models with idealized setups similar to those used in this study have been used to investigate the fate of buoyant coastal discharges in the near‐ and far‐field with or without wind‐driven dynamics (Cole & Hetland, 2016; Fong & Geyer, 2001; Hetland, 2005; Jurisa & Chant, 2013; Yankovsky & Chapman, 1997). Model simulations with realistic bathymetry and field conditions have also been used to study plume spreading (Hetland & MacDonald, 2008, 2010) and frontal processes similar to liftoff (Akan et al., 2018; Ralston et al., 2010, 2017; Wang et al., 2015, 2017). These studies examined the salinity structure of the frontal processes of the Merrimack, Columbia, and Hudson River estuaries, but the relationship between the salinity structure and the water surface elevation was not investigated. Water surface elevation changes at the same time as salinity structure changes were shown by McCabe et al. (2009) in their modeling study of the mouth of the Columbia River, but the changes were not investigated in detail. Several of these studies highlighted the importance of high grid resolution near the river mouth. Our study uses a high‐resolution model of a generalized river to determine how discharge, *α*, and *R*_*A*_ affect the location and water surface signal of liftoff.

In this paper, we examine the relationship between the physical parameters of the river/ocean system and the liftoff process to evaluate if liftoff has a detectable water surface signal that could be used to estimate discharge. We derive theoretical expressions for the liftoff length and ridge height that depend on discharge, shelf slope, and the river mouth width to depth aspect ratio (section 3). An idealized numerical model (section 4) is used to reproduce the dynamics of liftoff for a range of shelf slopes, aspect ratios, and discharge values including when the salt wedge is present and when liftoff occurs outside the river mouth. Modeled estimates of liftoff lengths and ridge heights are compared to predictions by the equations presented in section 3, and momentum balances are examined to understand why a ridge forms (section 5). In section 6 we discuss two important processes, plume spreading and tides, that influence the liftoff process and may impact its detectability. We also compare the magnitude of the predicted liftoff water surface expression to the resolution of the upcoming SWOT altimeter in order to gauge the feasibility of a remote algorithm based on this approach. Lastly, we present our final conclusions and suggestions for future work (section 7).

## 3 Theory

Here we present derivations for the liftoff length and ridge height in terms of *Q*, *b*_0_, *h*_*s*_, *α*, and a spreading parameter *κ*. All of these variables can be estimated or measured directly for most rivers except *κ*. We determine the range of values for *κ* in section 6.2.

### 3.1 Liftoff Length

We derive an equation for *L*_lo_ based on the assumption that the plume spreads on both sides at the speed of a gravity current, $\sqrt{g^{\prime }h_{1}}$. The plume width, *b*, varies according to ${ū}_{1}db/dx=2\sqrt{g^{\prime }h_{1}}$, which yields

(9) $$\frac{db}{dx}=\frac{\kappa }{Fr_{1}},$$

where *κ* is 2 for a surface‐trapped plume (Hetland, 2010; Poggioli & Horner‐Devine, 2018). When the plume is not trapped at the surface, but instead attached to the bottom until the liftoff location, *κ* is between 0 and 1 (Poggioli & Horner‐Devine, 2018). As the plume propagates away from the mouth, the depth increases, the cross‐sectional area of the plume increases, and *Fr*_1_ decreases toward 1. To capture this, the Froude number can be expressed in terms of the cross‐sectional area, *A*, and depth *h*_1_ as

(10) $$Fr_{1}=\frac{Q}{A\sqrt{g^{\prime }h_{1}}}.$$

To account for the variation in *A* with *x*, we approximate the plume cross section as a rectangle of width *b* and allow *h*_1_ to increase linearly from *h*_*s*_ at the mouth according to the shelf slope *α*, *h*_1_=*h*_*s*_+*αx*. Thus, the area is

(11) $$A=b(h_{s}+\alpha x).$$

Substituting Equations 10 and 11 into Equation 9 and integrating from *b*_0_ to *b* yields an expression for the axial variation of the plume width in terms of the freshwater Froude number.

(12) $$\frac{b}{b_{0}}=\exp\left[\frac{\Gamma }{Fr_{f}}(h_{*}^{5/2}-1)\right]$$

Here, *h*_∗_=1+*αx*/*h*_*s*_ is the dimensionless plume thickness before liftoff and Γ=2*κ*/(5*αR*_*A*_) is a parameter accounting for the spreading rate, shelf slope, and river mouth aspect ratio. Note that variations in the surface elevation, which are typically less than 2% of the flow depth (see Figure 6) are ignored in this formulation. At liftoff *Fr*_1_=1, *x*=*L*_lo_, and *Q* in Equation 10 can be written in terms of *Fr*_*f*_ which gives

(13) $$Fr_{f}={\left(1+\frac{\alpha L_{\text{lo}}}{h_{s}}\right)}^{3/2}\left[\exp\left\{\frac{\Gamma }{Fr_{f}}\left({\left(1+\frac{\alpha L_{\text{lo}}}{h_{s}}\right)}^{5/2}-1\right)\right\}\right].$$

Equation 13 provides an implicit expression relating *L*_lo_ to *Fr*_*f*_. For a known *Fr*_*f*_, Equation 13 can be solved using numerical methods to estimate *L*_lo_. If we consider the case where plume spreading is negligible by setting *κ* to zero, then Equation 13 reduces to

(14) $$Fr_{f}={\left(1+\frac{\alpha L_{\text{lo}}}{h_{s}}\right)}^{3/2},$$

which can be solved for the nondimensionalized liftoff length

(15) $$\frac{L_{\text{lo}}}{b_{0}}=\frac{1}{R_{A}\alpha }(Fr_{f}^{2/3}-1).$$

This equation is equivalent to Equation 7, which was derived by assuming a critical depth and zero offshore spreading (Geyer & Ralston, 2011). In section 6.2 we show that spreading is low when *α* is large enough such that Γ approaches zero. In this case, Equation 15 provides a good estimate of *L*_lo_.

### 3.2 Ridge Height

We derive an equation for ridge height based on the steady one‐dimensional *x*‐momentum equation for a fluid element,

(16) $$ū\frac{dū}{dx}=-\;g\frac{d\eta }{dx}-\frac{C_{D}{ū}^{2}}{h}.$$

Equation 16 can be further simplified assuming a rectangular plume of area, A, (Equation 11), quadratic bottom drag with drag coefficient *C*_*D*_, and ū=Q/A. Substituting *b* from Equation 12 into Equation 16 gives an expression for the water surface slope in terms of the nondimensional depth

(17) $$\frac{d\eta }{dh_{*}}=\frac{Q^{2}}{A^{3}g}\frac{dA}{dh_{*}}-\frac{C_{D}h_{s}}{h\alpha g}\left(\frac{Q^{2}}{A^{2}}\right),$$

which can be integrated between the river mouth and the liftoff location to arrive at an expression for the ridge height, *h*_ridge_

(18) $$\frac{h_{\text{ridge}}}{h_{s}}=\frac{g^{\prime }}{g}\;Fr_{f}^{2}\left\{\int_{1}^{1+\frac{\alpha L_{\text{lo}}}{h_{s}}}\frac{\frac{\Gamma }{Fr_{f}}2.5h_{*}^{5/2}+1-\frac{C_{D}}{\alpha }}{h_{*}^{3}\exp\left[\frac{2\Gamma }{Fr_{f}}(h_{*}^{5/2}-1)\right]}\;dh_{*}\right\}.$$

When plume spreading is negligible (*κ*=0), the integral simplifies to

(19) $$\frac{h_{\text{ridge}}}{h_{s}}=\frac{1}{2}\frac{g^{\prime }}{g}\left[\left(1-\frac{C_{D}}{\alpha }\right)\left(Fr_{f}^{2}-Fr_{f}^{2/3}\right)\right].$$

The details of this ridge height derivation can be found in the Appendix and a schematic of the important parameters is shown in Figure 2.

## 4 Methods

### 4.1 Model Description and Configuration

In this study, we use the Regional Ocean Modeling System, ROMS (Shchepetkin & McWilliams, 2005), to investigate liftoff dynamics and water surface elevation changes with discharge. ROMS is a three‐dimensional, free surface, primitive equation ocean model using orthogonal curvilinear coordinates in the horizontal direction and S‐coordinates in the vertical direction. It solves finite‐difference approximations of the Reynolds‐averaged Navier‐Stokes equations using the Boussinesq and hydrostatic approximations (Shchepetkin & McWilliams, 2005, 2009). ROMS was run in a idealized river/ocean system similar to configurations used by Hetland (2005), Cole and Hetland (2016), and Qu and Hetland (2019). Even though the idealized setup used in this study is not directly validated against field measurements, similar studies with idealized ROMS configuration have been used to study near‐field plume spreading region of the Merrimack River along with validation against observations (Chen et al., 2009). Realistic ROMS model applications used to study near‐ and far‐field plume dynamics also have been compared to field measurements (Liu et al., 2009; Pan et al., 2014; Rong et al., 2014). These studies highlight the role of tides, surface waves, and winds to cause unsteady flow conditions near the river mouth. Unsteady flow conditions can also be due to episodic storms upriver. Although rivers often have unsteady flow conditions, here we target steady flow conditions to quantify the model output to analytically derived liftoff lengths and ridge heights. This section describes the model configuration and the physical parameters used in the numerical simulations.

**Figure 3:**
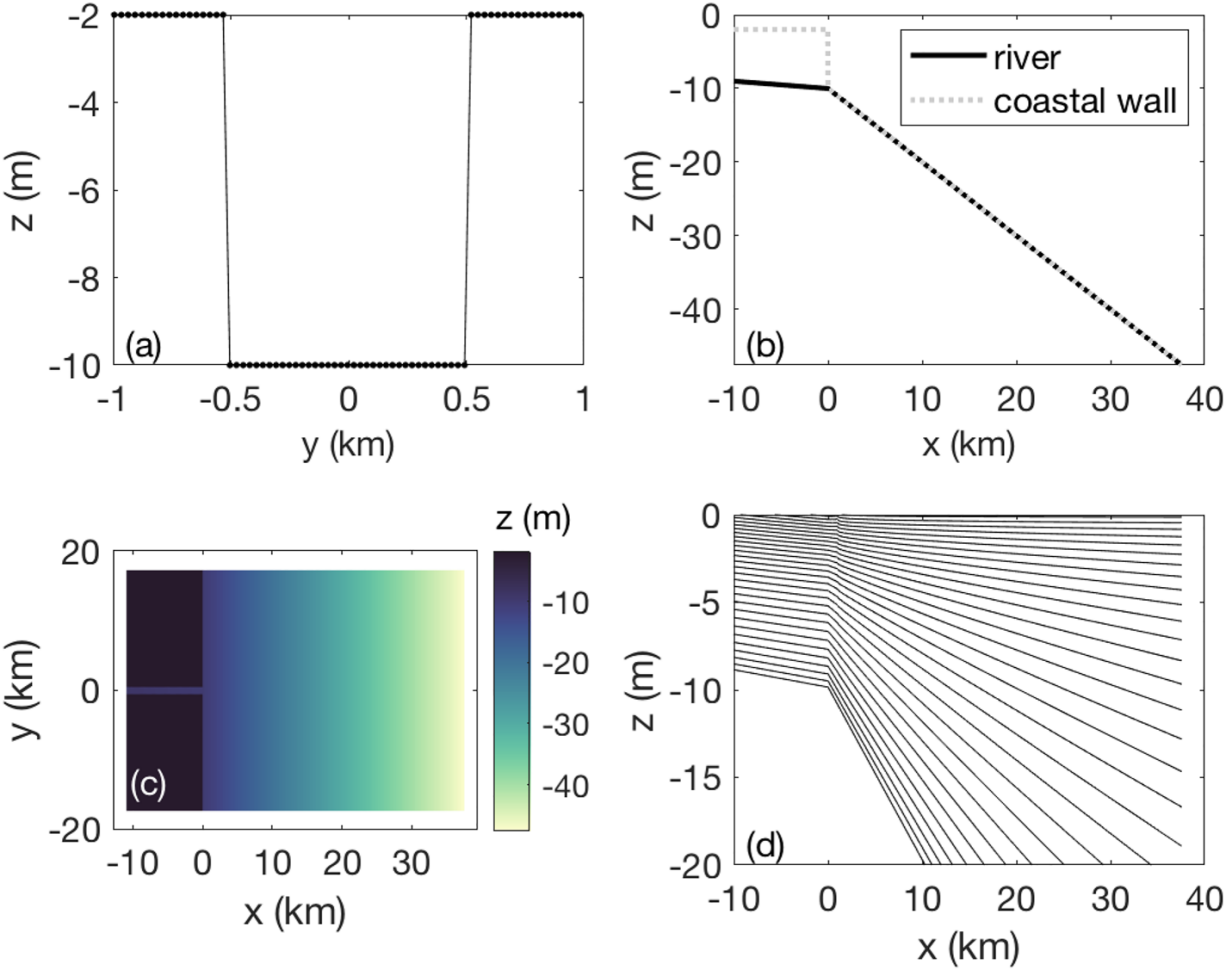
ROMS grid configuration: (a) Rectangular river mouth, (b) coastal wall, (c) bathymetry for high Froude number runs, and (d) S levels.

The model domain is 35 km in the alongshore direction and 48 km in the cross‐shore direction (Figure 3). It has a 10 km long, 1.025 km wide, rectangular river that is lengthened to 61 km for low‐discharge runs to contain the salt wedge. The river has a bottom slope of 0.0001 and empties into an ocean with a constant shelf slope, vertical coastal wall, and 30 S layers with resolution focused near the surface and the bottom (Figure 3d). Shelf slopes of 0.001, 0.002, and 0.005 were chosen for comparison with previous studies, for example, Yankovsky and Chapman (1997) and Poggioli and Horner‐Devine (2018). Sensitivity studies were conducted to determine the minimum resolution necessary to resolve the dynamics of plume liftoff and spreading near the mouth. The resolution in the cross‐shore direction varies from 25 to 200 m with the highest resolution at the river mouth. Water temperatures in the ocean and river were set to 25°C. This was done to constrain density differences to those due to salinity instead of salinity and temperature. The initial conditions were flow at rest and an ocean salinity of 32 psu. Chapman and Flather boundary conditions were used for sea surface elevation and barotropic velocities, and gradient boundary conditions were used for baroclinic flows, temperature, and salinity. Quadratic drag was assumed for the bottom with a drag coefficient, *C*_*d*_=0.003. A *k*−*ϵ* turbulence closure scheme was used as the vertical mixing algorithm. The time needed for the model to reach a steady state was determined to be 5 days as the liftoff lengths were constant in time after that point. Model runs were completed for three shelf slopes, three shoreline depths, and 10 discharge values characterized by their freshwater Froude numbers, as shown in Table 1.

### 4.2 Analysis

The model output was analyzed differently during low and high discharge. The salinity structure was used to calculate the salt wedge length during low discharge and the liftoff length during high discharge. The water surface slope change was estimated for all conditions at the liftoff location and additionally at the river mouth during high‐discharge conditions. The plume spreading parameter was calculated during high‐discharge conditions from vertical cross sections of salinity between the river mouth and the liftoff location.

#### 4.2.1 Low Discharge

During low‐discharge conditions liftoff is in the channel and the distance between the river mouth and the liftoff location is the salt wedge length, *L*_sw_ (Figure 1a). Liftoff dynamics simulated for *Fr*_*f*_ between 0.1 and 0.5 are further investigated by examining changes in the salt wedge length and the water surface elevation.

**Table 1:** ROMS Model Run Parameters

*α*	*h*_*s*_(*m*)	*Fr*_*f*_
0.001	10	low
0.001	10	high
0.002	10	high
0.005	10	high
0.005	5	high
0.005	15	high

*Note*. Low freshwater Froude numbers were 0.1, 0.2, 0.3, 0.4, and 0.5. High freshwater Froude numbers were 1.2, 2, 3, 4, and 5.

The salt wedge length is calculated as the distance between the river mouth and the location upstream where the midriver salinity in the bottom S layer decreases below 2 psu, which indicates that the water in that layer is predominantly freshwater. Sea surface height, *η*, averaged over the final 2 days of model simulation is used to calculate the surface slope, $\frac{d\eta }{dx}$, over 10 km upriver and downriver of the toe of the salt wedge. The surface slopes above and below the toe of the salt wedge are calculated over 10 km except for the *Fr*_*f*_=0.1 and *Fr*_*f*_=0.5 flow cases. For the lowest flow case (*Fr*_*f*_=0.1), the toe of the salt wedge is less than 10 km from the edge of the model grid; therefore, the slope upriver of the toe is calculated between the toe and the model grid edge. For the highest flow case (*Fr*_*f*_=0.5), the toe of the salt wedge is less than 10 km from the river mouth; therefore, the slope downstream of the toe is calculated along the entire distance between the toe and the river mouth.

#### 4.2.2 High Discharge

During high‐discharge conditions liftoff is outside the river mouth (Figure 1b) and the model output is first averaged over the final 2 days of simulation and then examined for different discharge, *α*, and *R*_*A*_ values. The liftoff length is calculated as the distance between the river mouth and the location of the maximum gradient of the salinity in the bottom S layer at the same alongshelf location as the middle of the river. The surface slope signature is quantified as the slope change between upriver and seaward of the mouth, but additionally the slope change at the ridge peak is calculated. The ridge height is calculated as the height difference between the offshore peak water level and the water level at the river mouth.

#### 4.2.3 Plume Spreading

Plume spreading dynamics are investigated in section 6.2 where we focus on the spreading parameter *κ*, which influences the liftoff length (Equation 13) and the ridge height (Equation 18). To calculate *κ* from the ROMS output the shape of the plume is found by determining which grid cells are in the plume at each location between the river mouth and the liftoff location. Grid cells are evaluated in each alongshore vertical salinity cross section. Example alongshore vertical salinity cross sections are shown in Figures 12e and 12f. Grid cells are considered to be in the plume if their salinity value is below a salinity threshold. The salinity threshold is set for each vertical cross section as the salinity where the sum of the volumetric freshwater flux in the plume grid cells is 85% of the total freshwater flux in that vertical cross section. The plume cross‐sectional area, *A*_plume_, and average plume velocity, *U*_plume_, are then calculated for the grid cells in the plume. The width of the spreading plume, *b*, is calculated as *A*_plume_ divided by the depth. The plume Froude number is calculated following Equation 1 as $Fr_{\text{plume}}=\frac{U_{\text{plume}}}{\sqrt{g^{\prime }h}}$ where *h* is the water depth. The plume spreading parameter, *κ*, is then calculated as $Fr_{\text{plume}}\frac{db}{dx}$ where *Fr*_plume_ is calculated for every vertical cross section between the mouth and the liftoff location and then averaged, d*b* is the width change of the plume between the mouth and the liftoff location, and d*x* is the distance between the mouth and the liftoff location.

## 5 Results

In this section the dependence of the liftoff process on discharge, *α*, and *R*_*A*_ is presented for both low and high discharge conditions. Modeled estimates of *L*_lo_ and *h*_ridge_ are compared to the analytical predictions (Equations 13 and 18) derived in section 3.

### 5.1 Low Discharge

**Figure 4:**
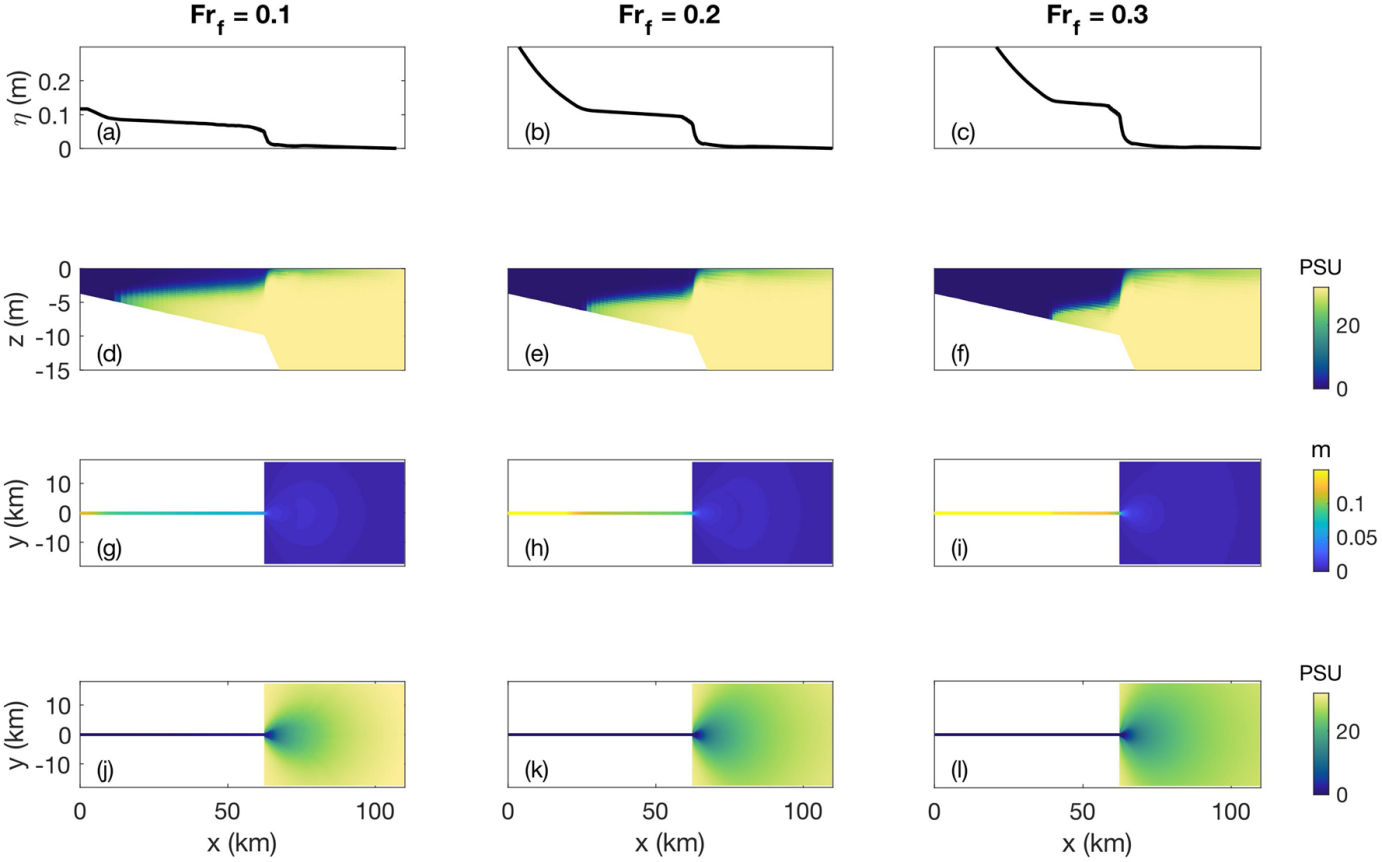
Low‐discharge examples of the water surface elevation and salinity structure for *α*=0.001 and *R*_*A*_=103: (a)–(c) Side view line plots of water surface elevation at the location of the middle of the river. (d)–(f) Side view cross sections of salinity with depth at the location of the middle of the river. (g)–(i) Plan view images of the water surface elevation. (j)–(l) Plan view images of the surface salinity.

**Figure 5:**
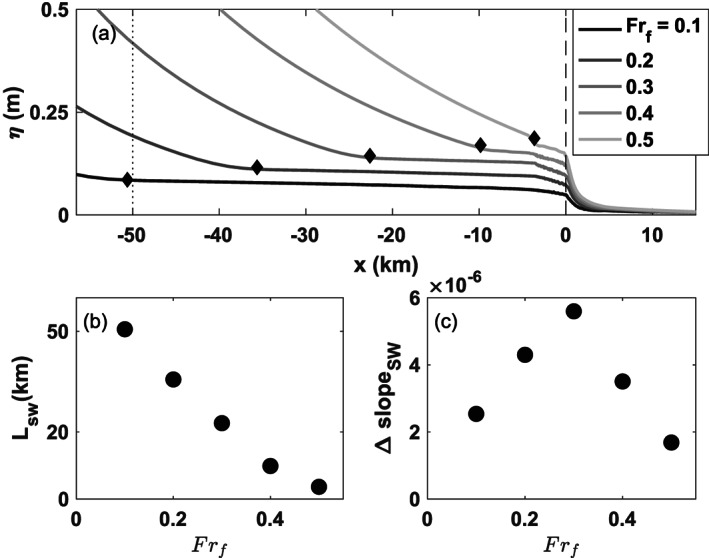
(a) Sea surface elevation, *η*, during low discharge for *F* *r*_*f*_ from 0.1 to 0.5 with the toe of the salt wedge marked by a diamond, the river mouth marked with a vertical dashed line, and the location where the five water surface elevations are distinct by 0.1 m marked with a dotted line. (b) Salt wedge length as a function of *F* *r*_*f*_. (c) Water surface slope change at the toe of the salt wedge.

Model output confirms that when *Fr*_*f*_<1 the salt water intrudes up through the mouth and liftoff occurs upstream in the river channel (Figures 4d–4f). As discharge increases, the higher flow pushes the salt wedge closer to the mouth, decreasing *L*_SW_ (Figures 4d–4f, 5a, and 5b). These findings are consistent with Poggioli and Horner‐Devine (2015) who explored the details of the relationship between *L*_SW_, discharge, and channel geometry. In this study we did not conduct simulations for different channel geometries at low discharge but instead focused on the water surface signal changes with discharge.

The water surface signal associated with liftoff when *Fr*_*f*_<1 is a decrease of the water surface slope at the toe of the salt wedge (Figures 4a–4c). This water surface slope change moves closer to the mouth as the discharge increases and *L*_SW_ decreases (Figures 5a and 5b). The slope of the upstream M1 backwater curve increases with discharge as expected for a gradually varied flow profile upstream of *x*=*L*_SW_ (Figure 5a). Downstream of liftoff the water surface slope is small. Physically, this occurs because the interfacial drag coefficient between the freshwater and the salt wedge is typically on the order of 10 times smaller than the drag coefficient of the river bottom. For increasing discharge, the difference between the M1 slope upriver of liftoff and the slope downriver of the liftoff location increases until *Fr*_*f*_=0.3 where it decreases due to the short length of the salt wedge (Figure 5c).

**Figure 6:**
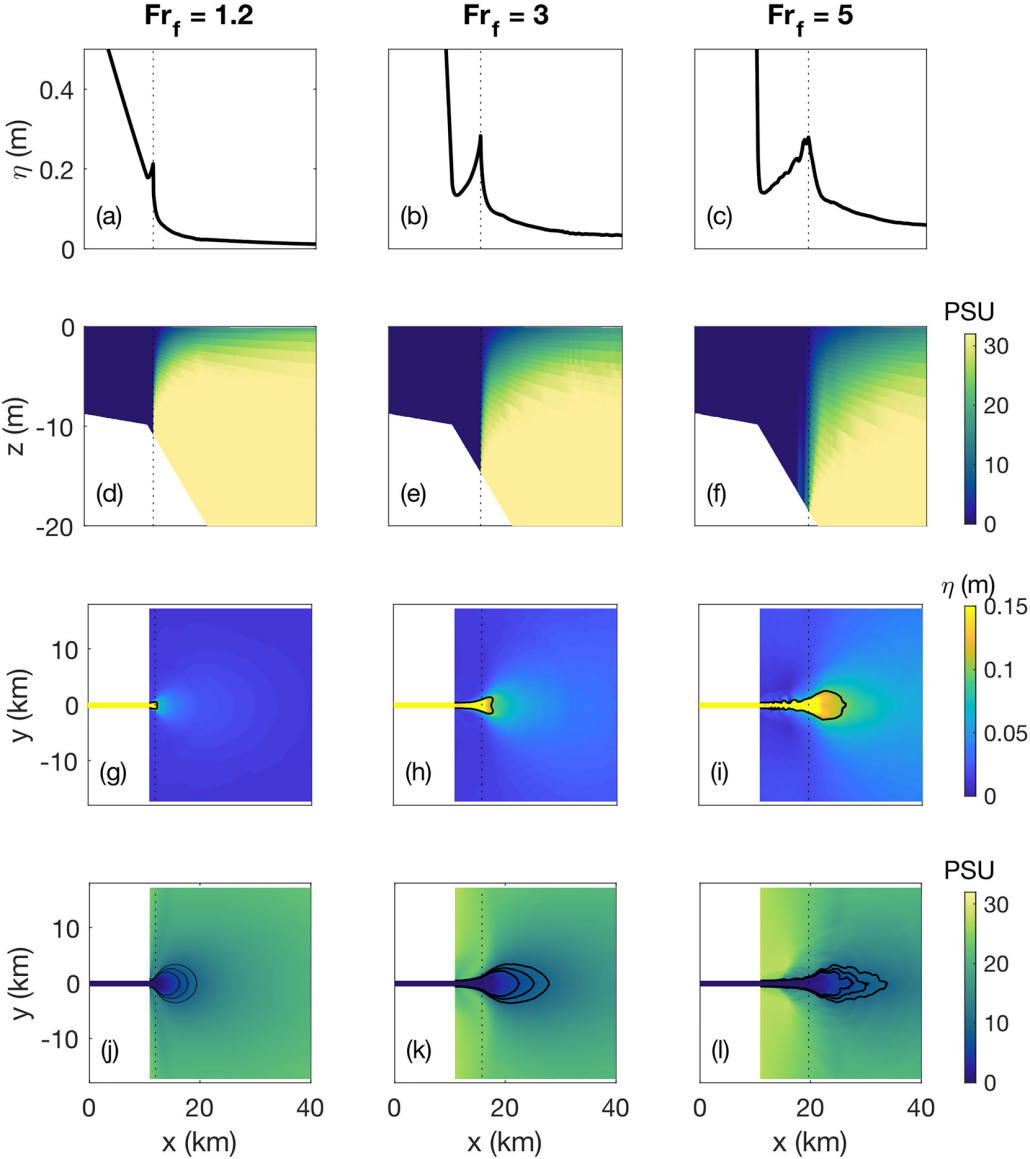
High discharge examples of the water surface elevation and salinity structure for *α*=0.001 and *R*_*A*_=103. The liftoff location is marked with a dotted line. (a)–(c) Side view line plots of water surface elevation at the location of the middle of the river. (d)–(f) Side view images of salinity with depth at the location of the middle of the river. (g)–(i) Plan view images of the water surface elevation with the 0.1 m elevation contour plotted as a black line. (j)–(l) Plan view images of surface salinity with the 6, 8, and 10 psu contours plotted as a black line.

Another water surface slope change occurs at the mouth of the river (Figure 5a) where the salinity structure changes to a laterally spreading thin surface plume (Figures 4d–4f and 4j–4l). This sharp change is primarily due to the plume spreading alongshore because it is no longer constrained by the river channel (Figures 4j–4l). The dramatic slope change may be a consequence of the square edges of the modeled river mouth, which are more abrupt than natural river mouths. After the dramatic slope change at the river mouth, the water surface elevation shows no structure offshore (Figures 4g–4i), which is in contrast to what is seen during high discharge (Figures 6g–6i) and discussed in section 5.2.

### 5.2 High Discharge

**Figure 7:**
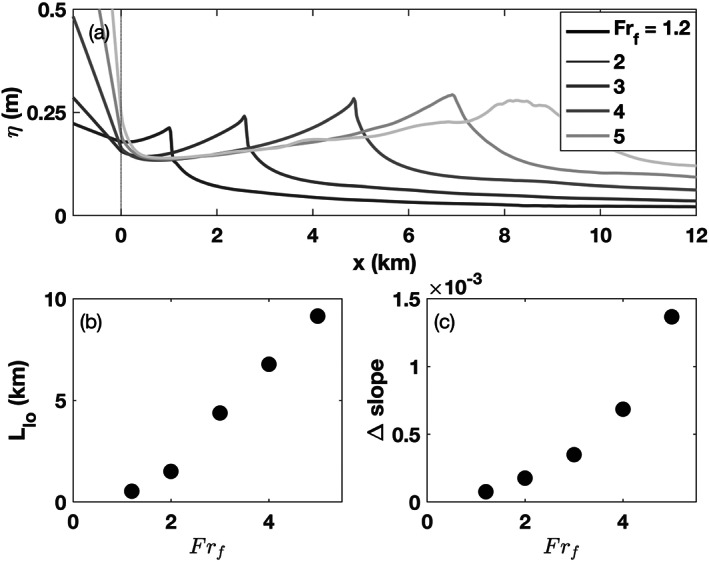
High discharge with *α*=0.001 and *R*_*A*_=103. (a) Sea surface height, *η*, versus cross‐shore distance for five freshwater Froude numbers from 1.2 to 5. The river mouth is marked with a dotted line. (b) Liftoff length as a function of *F* *r*_*f*_. (c) Water surface slope change at the river mouth.

When *Fr*_*f*_>1 the freshwater exits the river mouth as a jet that stays attached to the bottom of the shelf until it has slowed down enough to lift off (Figures 6d–6f). At the liftoff location it spreads laterally into an oval‐shaped plume and is confined to a near‐surface layer (Figures 6d–6f and 6j–6l). With an increase in discharge, the size of the plume increases and the freshwater stays attached to the bottom further offshore, thus leading to an increase in *L*_lo_ (Figures 6d–6f, 6j–6l, and 7b).

In high‐discharge conditions, the water surface forms a three‐dimensional ridge outside the river mouth, which increases in size with discharge (Figures 6g–6i). The ridge forms on the water surface as the result of a positive surface slope between the mouth and the liftoff location and the dramatic decrease of the water surface slope at liftoff. The formation of this positive slope seaward of the mouth will be discussed further by examining the conservation of momentum in section 5.5.

As discharge increases, the ridge peak increases in height and moves further from the mouth (Figure 7a). At the highest discharge of *Fr*_*f*_=5 the ridge becomes unstable in our simulations as indicated by small‐scale fluctuations in the cross‐shelf evolution of the ridge, suggestive of a dynamical steady state (Figure 7a). The distance between the mouth and the ridge peak is the ridge length, *L*_ridge_, which for the purposes of this paper will be considered equal to *L*_lo_ because the peak of the ridge is directly above the liftoff location (Figures 6a–6f).

Upriver of the mouth the water surface slope is an M2 profile (Sturm, 2010), whose slope increases with discharge (Figure 7a). The slope change between the upriver M2 profile and the positive slope seaward of the mouth also increases with discharge (Figure 7c).

### 5.3 Liftoff Length Dependence on *α* and *R*_*A*_

**Figure 8:**
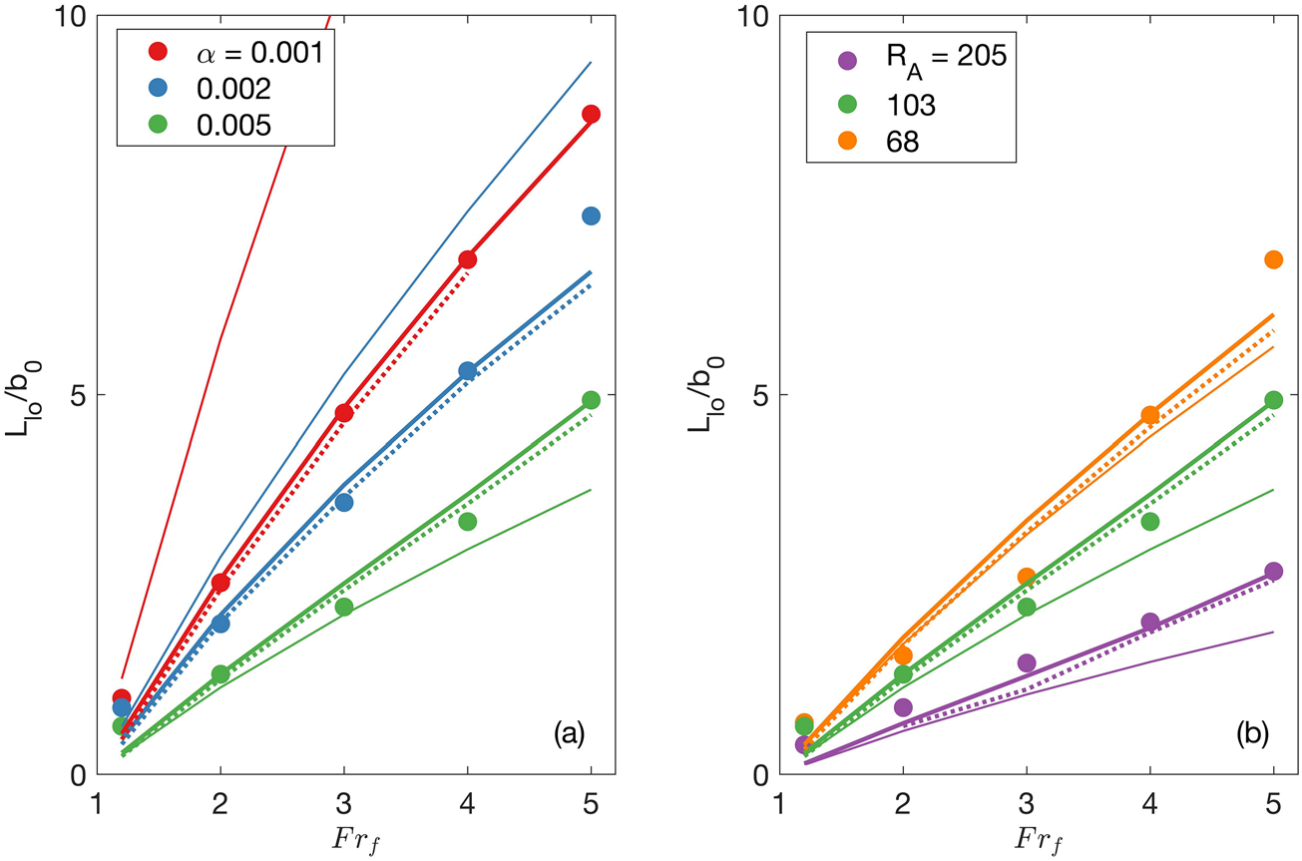
Normalized liftoff lengths versus freshwater Froude number for (a) three shelf slopes (*R*_*A*_=103) and (b) three river mouth aspect ratios all with a shelf slope of *α*=0.005. Filled circles are ROMS estimates, thick solid lines are calculated with Equation 13 and *κ* is optimized to minimize the difference with the ROMS estimates. Thin solid lines are calculated with Equation 15 where *κ*=0, and dashed lines are calculated with the Poggioli and Horner‐Devine (2018) model using the optimized *κ* values.

The dependence of the liftoff length on discharge, *α* and *R*_*A*_ is explored in Figure 8, which shows dimensionless liftoff lengths computed from ROMS output compared with analytical model predictions of Equations 13 and 15, and the Poggioli and Horner‐Devine (2018) hydraulic model. Note that the Poggioli and Horner‐Devine (2018) model returns no prediction for some high *Fr*_*f*_ values because no hydraulic solution exists. For all values of *α* and *R*_*A*_, liftoff occurs further offshore as *Fr*_*f*_ increases, consistent with the expectation that liftoff is further away from the mouth as discharge increases (Jones et al., 2007; Geyer & Ralston, 2011; Poggioli & Horner‐Devine, 2018; Safaie, 1979). The river width is approximately 1 km so the simulated dimensional liftoff lengths are between 1 and 8 km.

The relationship between *L*_lo_ and *Fr*_*f*_ also depends on shelf slope and river mouth aspect ratio. Higher values of *α* result in shorter liftoff lengths for all freshwater Froude numbers because the increase in cross‐sectional area results in more rapid flow deceleration. The inverse dependence between liftoff length and shelf slope is consistent with prior models (Geyer & Ralston, 2011; Poggioli & Horner‐Devine, 2018; Safaie, 1979). The river mouth aspect ratio also influences the liftoff location; a higher aspect ratio results in a smaller *L*_lo_ as predicted by Safaie (1979), Jones et al. (2007), and Geyer and Ralston (2011). A higher aspect ratio results in a larger bottom area relative to the plume volume, which means an increase in the effective bottom drag on the plume. The elevated bottom drag decelerates the plume quickly and the plume lifts off close to the river mouth. It is important to note that in our simulations the channel width has been held fixed and *R*_*A*_ is only varied by changing *h*_*s*_. We expect, however, that the same result would be obtained by changing the channel width because a larger channel width would also mean a larger bottom area relative to the plume volume. Once the channel width is larger than the Rossby radius the flow separates from the channel wall within the estuary. This process will further influence the dynamics near the river mouth, but consideration of Earth's rotation is beyond the scope of the present study.

Predictions of *L*_lo_ based on Equation 13 show good agreement with the ROMS results and the Poggioli and Horner‐Devine (2018) hydraulic model for the full range of *Fr*_*f*_, *α*, and *R*_*A*_ values (Figure 8). In these predictions, the spreading rate *κ* is not known a priori. For each set of runs with a fixed *α* and *R*_*A*_, *κ* is determined by minimizing the error between Equation 13 and the ROMS predicted liftoff lengths for the range of *Fr*_*f*_ values. Thus, each thick solid line in Figure 8 was computed with a single value of *κ*, which ranges from −0.18 to 0.22. The *κ* values were also entered into the Poggioli and Horner‐Devine (2018) hydraulic model to compute the *L*_lo_ and the predictions agree well with those of Equation 13 (Figure 8). In section 6.2 we determine the spreading rate from the ROMS salinity fields directly and show that the values of *κ* from these fits are consistent with the observed spreading.

Predictions of *L*_lo_ for the case with no plume spreading (*κ*=0, Equation 15) are also shown in Figure 8. They increase with *Fr*_*f*_, decrease for higher values of *α* (Figure 8a) and *R*_*A*_ (Figure 8b). The liftoff lengths predicted with Equation 15 match well with those calculated from the ROMS output for the case when the shelf slope is 0.005 (Figure 8b). However, the agreement breaks down for lower values of the shelf slope (Figure 8a). This suggests that liftoff is controlled by depth variation on steep shelves and spreading has a secondary influence. Both appear to be important on gentle shelves.

### 5.4 Ridge Height Dependence on *α* and *R*_*A*_

**Figure 9:**
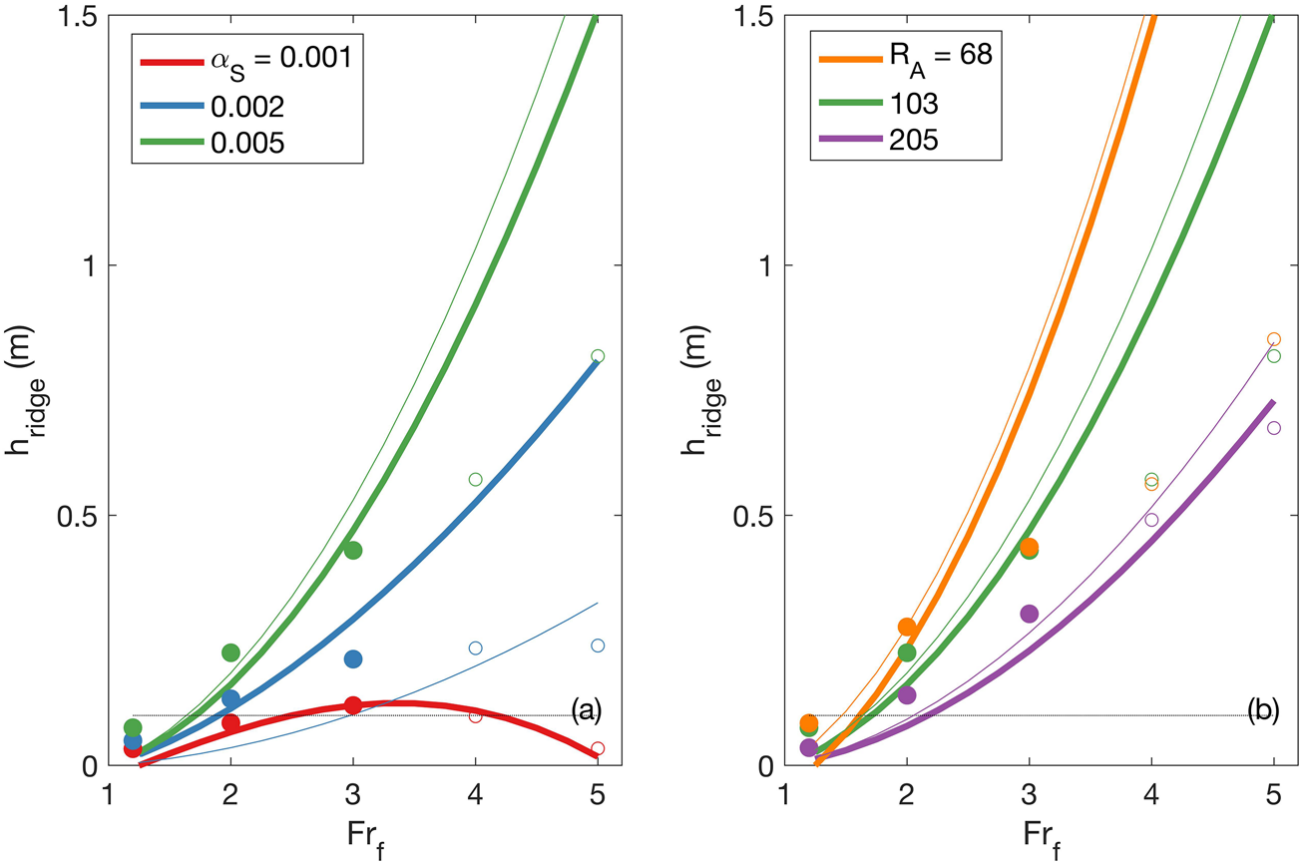
Ridge heights for (a) three shelf slopes (*R*_*A*_=103) and (b) three channel aspect ratios for a shelf slope of 0.005. Filled circles are ROMS estimates for *F* *r*_*f*_ ≤ 3 and open circles are ROMS estimates for *F* *r*_*f*_ ≥ 3. Thick lines were calculated with Equation 18 and thin lines were calculated with Equation 19 where *κ*=0. The predicted SWOT vertical accuracy of 0.1 m is shown as a dotted line.

The dependence of the ridge height on discharge, *α*, and *R*_*A*_ is explored in Figure 9. For almost all values of *α* and *R*_*A*_ the ridge height increases monotonically as discharge increases. Ridge height decreases for freshwater Froude numbers above 3 and *α*=0.001 because the water surface elevation at the mouth increases (Figure 7a) and we have defined ridge height as the height difference between the offshore peak and the water level at the river mouth. The ROMS output shows that ridge height depends more strongly on shelf slope than aspect ratio (Figure 9). Both the ROMS output and Equation 18 predict ridge height will increase with shelf slope for all freshwater Froude numbers (Figure 9a). When the aspect ratio is small (*R*_*A*_ ≤ 103) and *Fr*_*f*_ ≥ 3, Equation 18 significantly overpredicts the ridge height determined from the ROMS output (Figure 9). We expect that this is because the size and shape of the plume base that makes contact with the seafloor deviates more from the rectangular planform assumed in the derivation of Equation 18 when *R*_*A*_ is low and *Fr*_*f*_ is high. As a result, the bottom drag felt by the plume in the bottom‐attached region is higher than that predicted by the analytical model, and the ridge height is overpredicted. This is further explored by examining the momentum balance in section 5.5.

Equation 19 is a simplified version of Equation 18 where *κ*=0. It agrees best with Equation 18 and the ROMS output for *α*=0.005 (Figure 9). It underestimates *h*_ridge_ when *α*=0.002 and predicts negative values when *α*=0.001. The negative values are not shown on Figure 9a. This highlights the importance of plume spreading when *α* ≤ 0.002, which will be discussed further in section 6.2.

### 5.5 Momentum Balance

**Figure 10:**
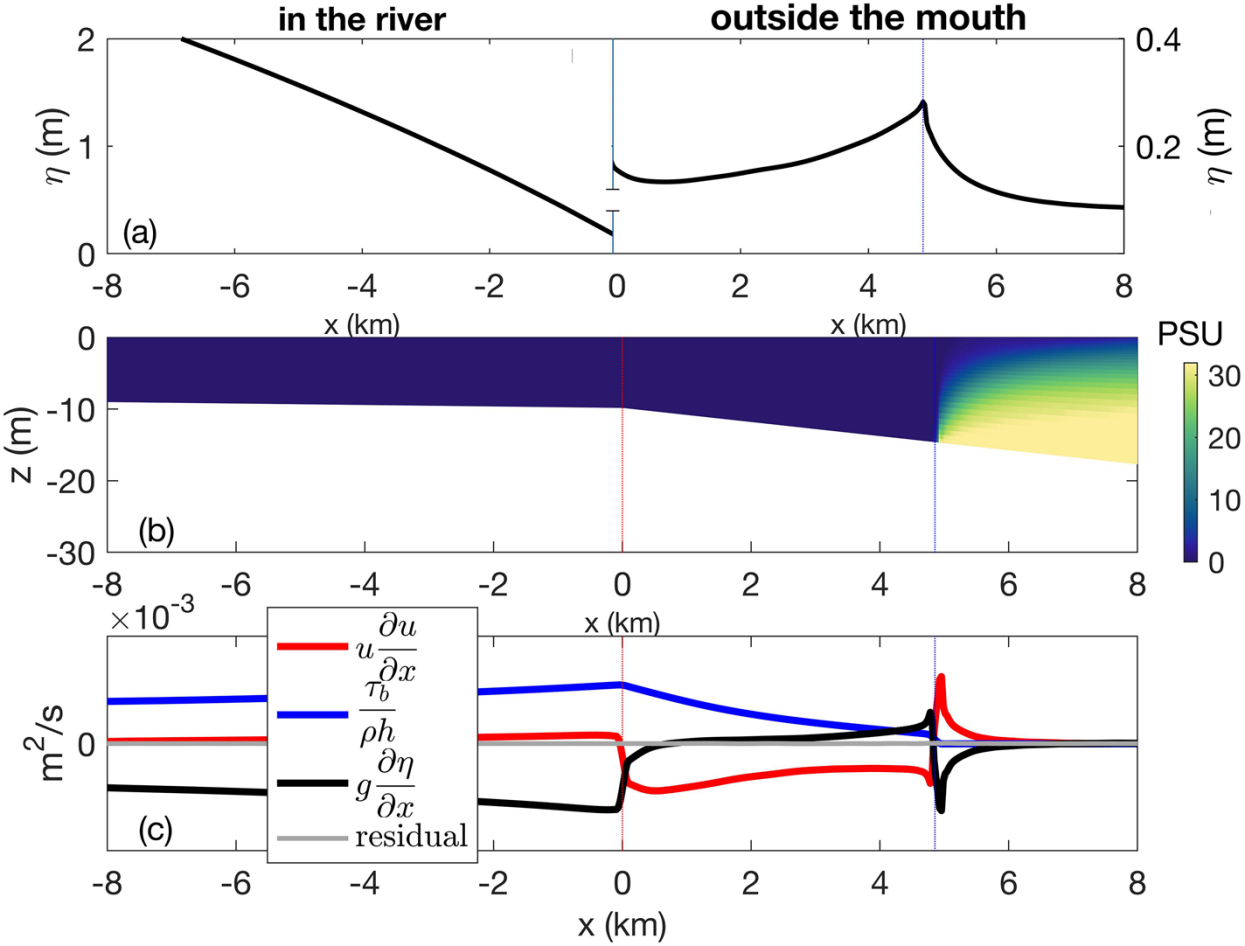
Water surface elevation profile, cross‐shore salinity cross section and *x*‐momentum terms for *F* *r*_*f*_=3, *α*=0.001, and *R*_*A*_=103. The river mouth is marked with a dotted red line and the liftoff location is marked with a dotted blue line. (a) Water surface elevation. Note that the scales for the surface elevation inside and outside the river mouth are different and indicated on the left and right *y* axes, respectively. (b) Side view of salinity. (c) Three dominant terms of the *x*‐momentum equation and their residual.

The mechanism responsible for ridge formation can be understood by examining the momentum balance terms based on the ROMS output. Here we consider a single high discharge run (*Fr*_*f*_=3, *α*=0.001, and *R*_*A*_=103) to illustrate the dynamics (Figure 10). The three dominant terms in the depth‐averaged *x*‐momentum balance are the advection, bottom stress, and pressure

(20) $$ū\frac{dū}{dx}+\frac{\tau_{b}}{\rho h}+g\frac{d\eta }{dx}=0$$

where ū is the depth averaged cross‐shore velocity and *τ*_*b*_ is the bottom stress. The residual of these terms is essentially zero (Figure 10c), confirming that the magnitudes of the other momentum terms are negligible. The momentum terms in Equation 20 are examined to understand the water surface elevation in the river, between the river mouth and the liftoff location, and seaward of the liftoff location. In this high‐discharge run there is no salt water in the lower reaches of the river and the momentum balance is primarily between the pressure and bottom stress terms. The water surface elevation profile displays the drawdown and M2 behavior predicted by hydraulic models (Sturm, 2010); the flow gets shallower and accelerates as it approaches the river mouth, resulting in a positive, increasing advection term (Figure 10c). The water surface slope and the corresponding pressure term are negative (Figures 10a and 10c). The bottom stress is increasing due to the accelerating velocity and its magnitude balances the advection and pressure terms (Figure 10c).

At the mouth, the depth increases and the water spreads laterally. As a result, the velocity decreases and the advection term switches sign suddenly, becoming negative (Figure 10c). Between the river mouth and the liftoff point the dominant balance is between the advection term, which slowly decreases in magnitude seaward, and the bottom stress term, which is positive and also decreases seaward as the velocity decreases (Figure 10c). The pressure term has to balance the advection and stress terms, resulting in a small but positive pressure term and a positive surface slope (Figures 10a and 10c).

At liftoff the bottom stress term drops to zero immediately as the plume loses contact with the bottom (Figures 10b and 10c). The plume layer thins rapidly and accelerates, causing the advection term to switch sign and increase to a local maximum immediately offshore of the liftoff point. These two changes require that the surface slope also changes sign, resulting in a peak in the water surface elevation at the liftoff location (Figures 10a–10c). Thus, the ridge is due to the deceleration of the flow in the region between the mouth and the liftoff point, which leads to an imbalance between the advection and bottom stress terms. As discharge increases, the advection term increases more than the bottom stress term, which leads to a larger imbalance and therefore a higher ridge (Figure 9).

Seaward of the liftoff point the momentum balance is between the pressure term and the advection term, since bottom stress is zero (Figure 10c). The rapid acceleration experienced by the plume at liftoff decreases seaward so the magnitude of the advection term and the compensating pressure term both decrease. Further from the mouth, all three terms decrease to near zero and the water surface elevation decreases to the surrounding water level (Figures 10a and 10c).

It is important to note that the momentum balance presented above is depth averaged, so interfacial stress in the plume offshore of the liftoff point is not evident. We expect that a full three‐dimensional momentum balance would indicate a dominant order balance between deceleration of the plume as demonstrated by a decrease in the horizontal advection term and the interfacial mixing and observed by Kilcher et al. (2012).

## 6 Discussion

Modeled liftoff lengths and ridge heights compare well with analytical solutions (Figures 8 and 9) presented in this study. Given the importance of the liftoff location on coastal morphology and surface signature, we further investigate the liftoff length dependence on *Fr*_*f*_ (section 6.1). In addition, we provide a physical description of the spreading parameter *κ*, and describe its dependence on the shelf slope (section 6.2). The role of barotropic tidal processes in changing the liftoff location and the ridge height is discussed in section 6.3. Finally, in section 6.4, we compare the magnitudes of the slope and elevation signals predicted by the numerical model with the predicted accuracy of the upcoming satellite altimeter SWOT to determine if SWOT has the potential to detect the liftoff location and use it to estimate discharge.

### 6.1 *L*_lo_ Comparisons

**Figure 11:**
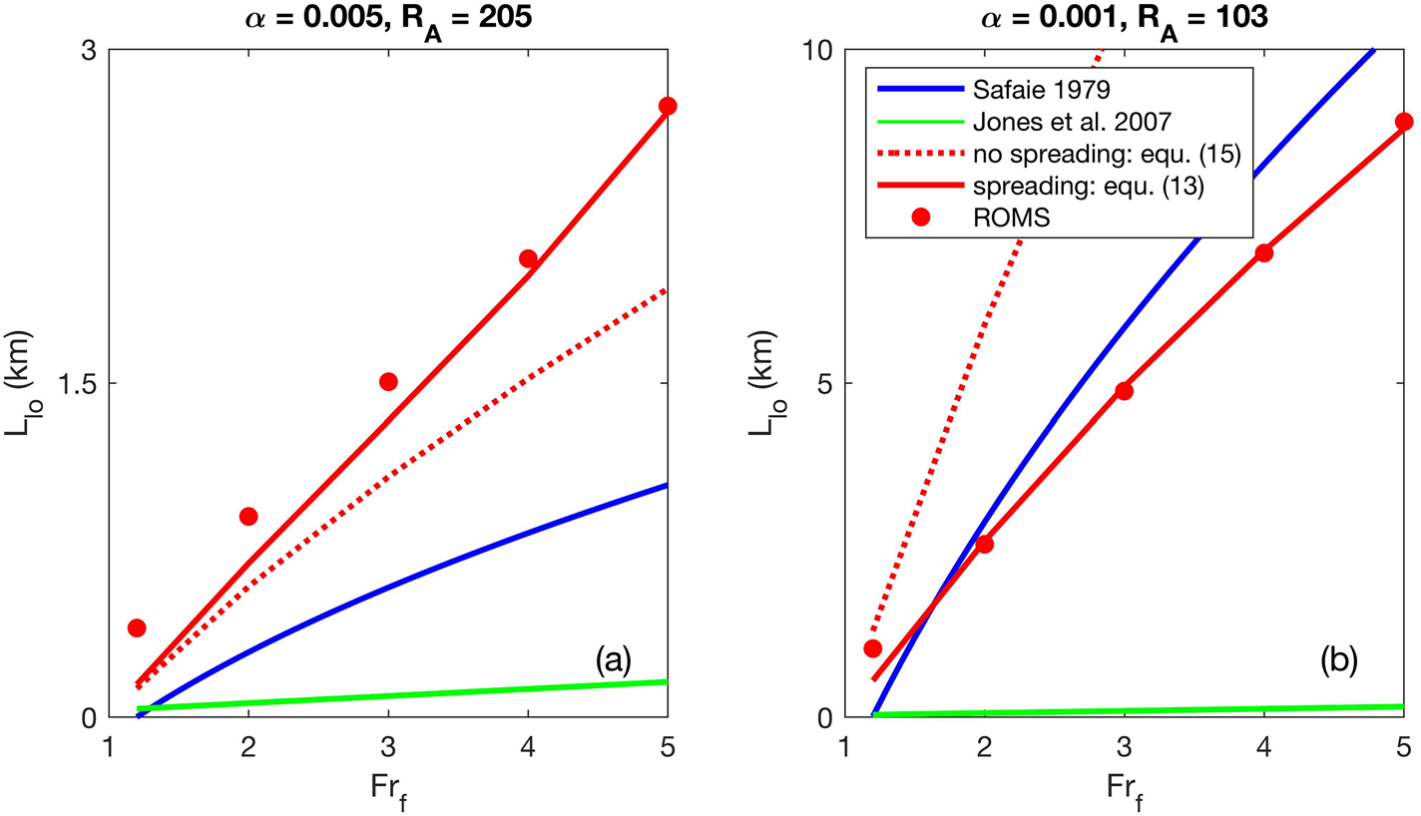
Liftoff length comparison of equation predictions to ROMS estimates for (a) *α*=0.005 and *R*_*A*_=205, and (b) *α*=0.001 and *R*_*A*_=103.

Two example comparisons of the liftoff lengths predicted by equations to ROMS liftoff lengths are shown in Figure 11. All of the equations predict *L*_lo_ will increase with *Fr*_*f*_ but the Jones et al. (2007) predictions are much lower than those of the other equations and of the ROMS estimates. This is most likely due to the fact that the Jones et al. (2007) equation has no dependence on shelf slope. The Safaie (1979) equation underestimates *L*_lo_ when *α* and *R*_*A*_ are high (Figure 11a) and overestimate *L*_lo_ when *α* and *R*_*A*_ are lower (Figure 11b). This behavior is consistent with our Equation 15 that does not include spreading, which indicates that the Safaie (1979) equation may not capture the physics of spreading correctly for all cases. This may be due to laboratory experimental constraints from which the equation was derived. Our Equation 13, which includes spreading, predicts liftoff lengths that are closest to the ROMS predictions.

**Figure 12:**
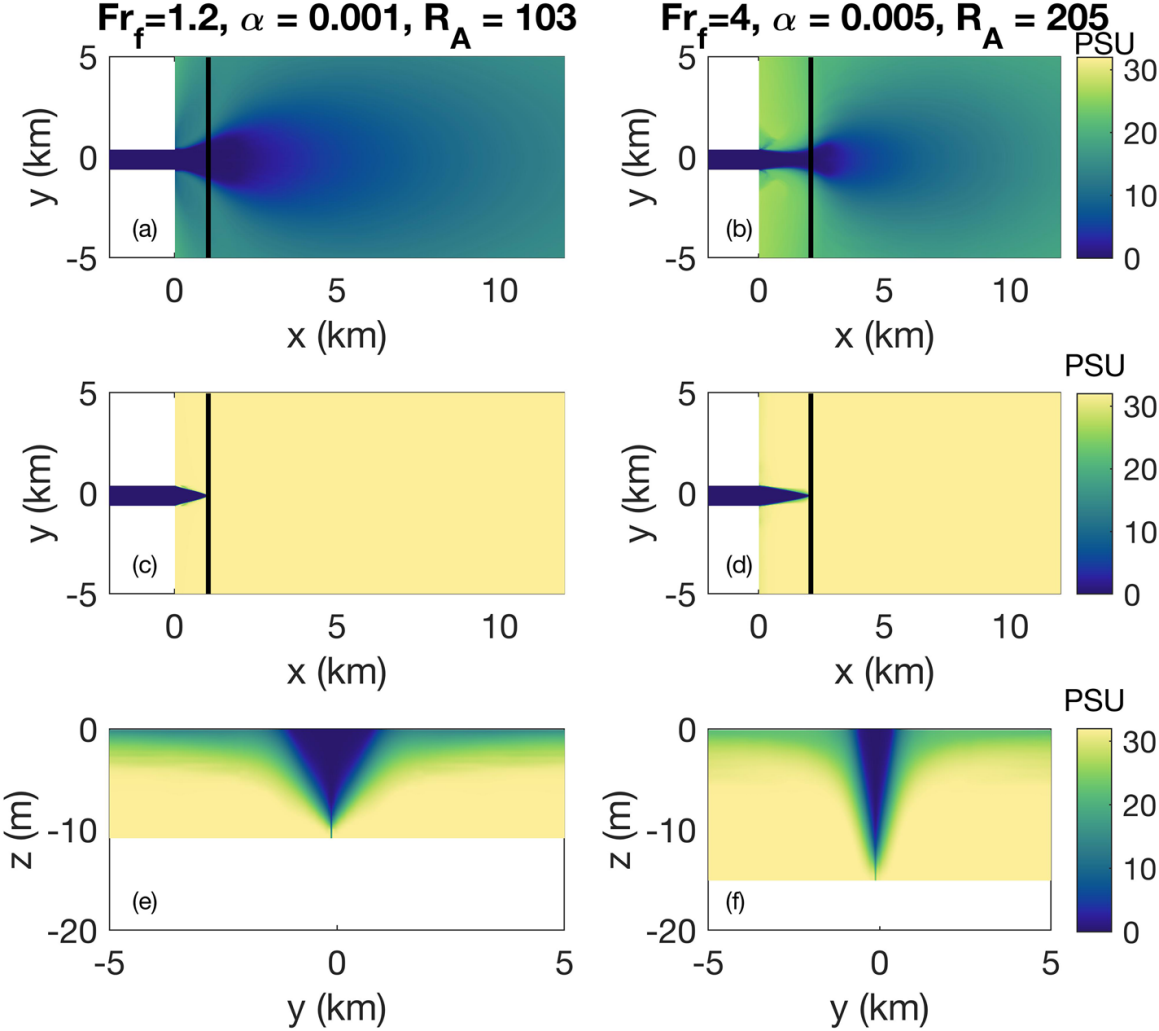
Salinity cross sections for the cases with a large *κ* (*F* *r*_*f*_=1.2,*α*=0.001,*R*_*A*_=103) and small *κ* (*F* *r*_*f*_=5,*α*=0.005,*R*_*A*_=205): (a, b) surface, (c, d) bottom, (e, f) vertical at the liftoff location. The liftoff location is marked with a black line on the surface and bottom cross sections.

### 6.2 Plume Spreading and Attachment

We observe that plume spreading depends on *α*, *R*_*A*_, and *Fr*_*f*_. Here, two contrasting examples are considered to further investigate the dependence on these parameters, focusing in particular on how spreading is represented by *κ*. For low freshwater Froude number (*Fr*_*f*_=1.2) and shelf slope 0.001, the plume starts spreading immediately after exiting the river mouth (Figure 12a); however, the attachment to the bottom narrows as the flow gets farther away from the river mouth (Figures 12c and 12e). At higher discharge (*Fr*_*f*_=4) and a steeper slope (0.005), the plume exiting the river mouth barely spreads before liftoff (Figures 12b and 12f). The analytical expressions derived in section 3 quantify this spreading behavior through the spreading parameter *κ* according to Equation 9. In section 5.3, we used *κ* as a fitting parameter in our analysis of liftoff length (Figure 8). Here, we describe the observed trends in *κ* and evaluate how it is related to the model simulated spreading.

**Figure 13:**
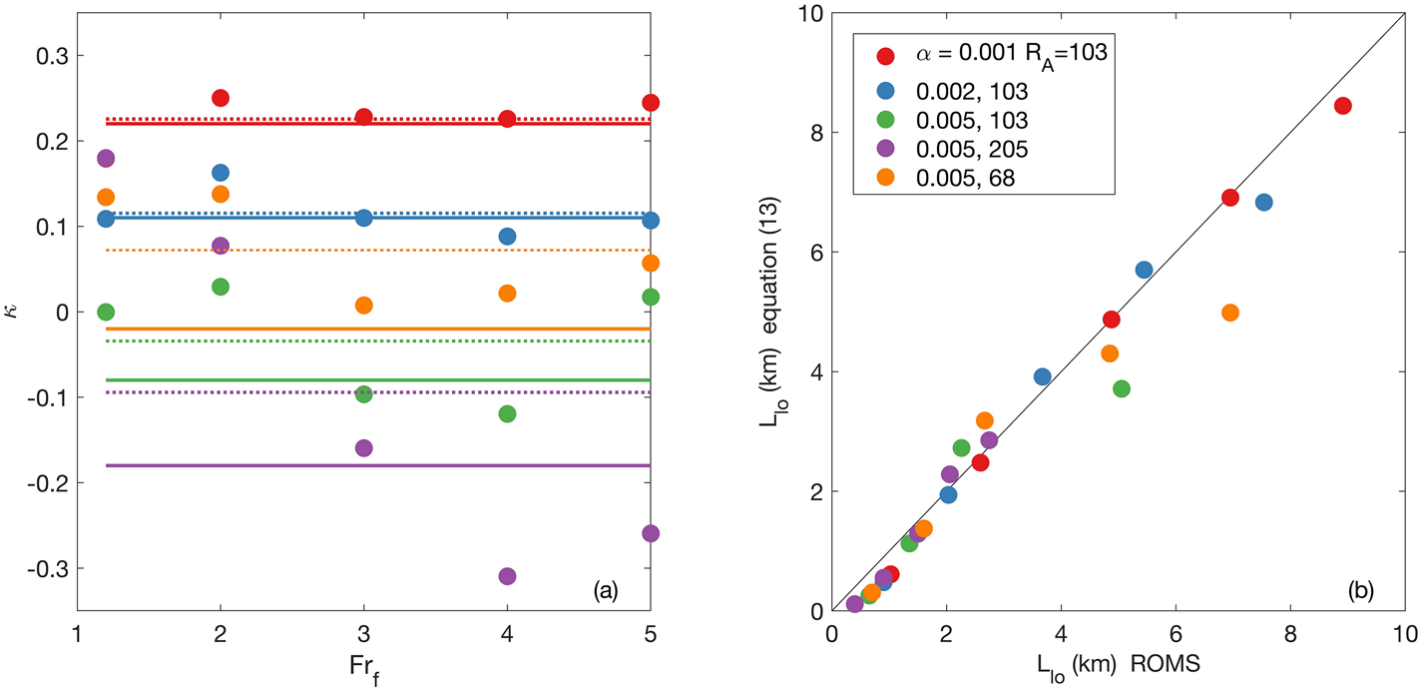
Estimations of the spreading parameter and its effect on *L*_lo_ calculations (a) *κ* as a function of *α*, *R*_*A*_, and *F* *r*_*f*_. Solid lines are the *κ* values determined by minimizing the difference between the liftoff lengths calculated with (13) and the ROMS estimates of *L*_lo_. Circles are calculated from the freshwater flux, dashed lines are averages of the circle values over *F* *r*_*f*_. (b) Liftoff lengths calculated with Equation 13 and *κ* from the freshwater flux calculation versus liftoff lengths estimated from the ROMS output.

The liftoff lengths shown in Figure 8 were calculated assuming *κ* does not vary with *Fr*_*f*_. The *κ* values used in the *L*_lo_ estimates were determined by minimizing the difference between the liftoff lengths calculated with Equation 13 and the ROMS estimates of *L*_lo_. The estimated *κ* values are shown on Figure 13a and suggest that *κ* increases as the shelf slope decreases for a constant aspect ratio. Furthermore, *κ* is negative for the steeper shelf slopes. The negative values, which indicate a narrowing plume, are a result of the approximation of the plume shape as a rectangle in Equation 11; the rectangular plume must narrow to compensate for the rapid deepening as it moves offshore.

In order to test whether the optimized *κ* values generated by the above fitting procedure correctly represent the physical spreading process, we compare them with an estimate *κ* derived from spreading observed using the model output. The methodology for estimating spreading rates and *κ* from the model output was described in detail in section 4.2.3. This analysis confirms that *κ* values derived from the observed spreading increases as the shelf slope decreases but also varies with *Fr*_*f*_ (Figure 13a). When the model derived *κ* values are averaged over *Fr*_*f*_ they agree well with the optimized values for the smaller shelf slopes (Figure 13a). However, the average *κ* values do not agree as well with the optimized values for the steeper shelf slopes due to higher variability with *Fr*_*f*_. The variability suggests there is a dependence of the spreading rate on *Fr*_*f*_. The dynamics of this dependence have not been investigated here but the apparent decrease in *κ* as *Fr*_*f*_ increases for the *α*=0.005 and *R*_*A*_=205 case is consistent with observations in near‐field plume spreading of a change from a convergent plume to a divergent plume (Yuan & Horner‐Devine, 2013). Despite the differences in the estimated *κ* values, the liftoff lengths calculated using *κ* values derived from the freshwater flux agree well with the liftoff lengths extracted from the ROMS output (Figure 13b).

### 6.3 Tidal Influence on the Ridge

Model results discussed so far do not take into account the role of barotropic tides, which change the dynamics of many coastal processes associated with riverine discharge (Liu et al., 2009; MacCready et al., 2009; Suanda et al., 2017). To gauge the importance of tides on liftoff dynamics and their surface signature, we conduct an additional simulation with a 1.5 m amplitude *M*_2_ semidiurnal tide (*α*=0.001, *h*_*s*_=10 m, and *Fr*_*f*_=2).

**Figure 14:**
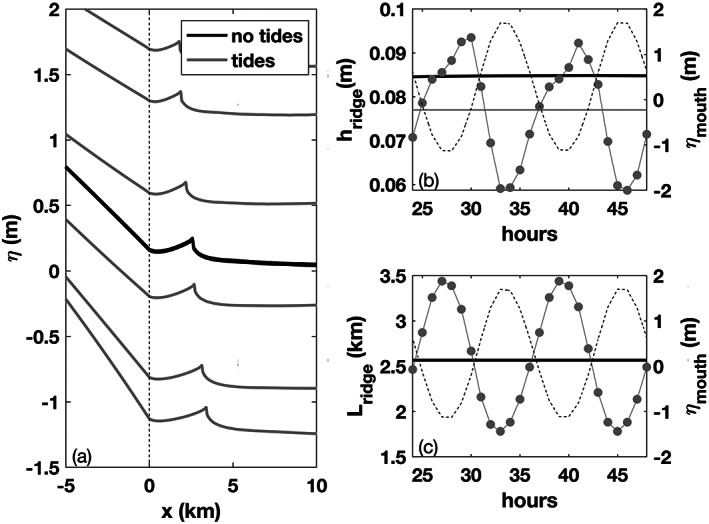
Tidal influence on the ridge after running the model for 24  hr. (a) Water surface elevation without tides (black) and six water surface elevation profiles over 6 hr of the tide (gray), (b) ridge height, *h*_ridge_, in gray and water surface elevation at the mouth, *η*_mouth_, as a dotted line. The solid black line marks *h*_ridge_ without the tide and the solid light gray line marks the average *h*_ridge_ with the tide. (c) Distance from the river mouth to the ridge peak, *L*_ridge_, in gray and *η*_mouth_ as a dotted line.

Tidal variability in the location and height of the ridge are shown in Figure 14. The ridge peak is closest to the mouth during high tide and farthest from the mouth during low tide (Figure 14c). Flood tide with an opposing current to the river flow effectively lowers the net offshore velocity and *Fr*_*f*_ in Equations 13 and 15. The lower *Fr*_*f*_ leads to a shorter *L*_lo_ and therefore reduces *L*_ridge_. Ebb tide has the opposite effect, amplifying the offshore current, effectively raising the *Fr*_*f*_ and increasing *L*_lo_ and *L*_ridge_. Ridge height is also modulated by tidal propagation; it is at a maximum on flood tide and minimum at high tide (Figure 14b). The highest ridge heights occur during peak tidal flood velocity when the tidal current opposes the river velocity leading to a significant velocity gradient between the river mouth and the liftoff location. This strong velocity gradient is manifested as a large advective acceleration term in Equation 16. Since the bottom stress term is decreasing due to spatial flow deceleration, a larger‐pressure term is needed to balance the advection term. The smallest ridge heights occur during slack high tide when the advection term is closer to balancing the bottom stress term. The maximum ridge height change due to the tide is 0.04 m, and the maximum distance that the peak moved over the tidal cycle is 1.7 km. On average, the tide lowers the height of the ridge from 0.085 to 0.077 m for this run (Figure 14b). This could be due to tidally induced mixing, tidal convergence, or the complex interaction between the phase of the tide and the time scale associated with the liftoff process and ridge formation.

These results show that while the tide does affect the ridge height and position, it does not eliminate the ridge from the surface. For lower discharge conditions (e.g., *Fr*_*f*_≈1) or large tidal amplitudes, the tidal signal may dominate the water surface elevation signal. A more detailed investigation of the influence of tides on the water surface signature of liftoff is left for future work.

### 6.4 Implications for SWOT Measurements of *Q* at the River Mouth

Satellite altimeters such as SWOT detect water surface elevation and water surface slope, which may be used in remote sensing estimates of discharge. Modeled water surface elevation and slope changes can be compared to the predicted accuracy of SWOT measurements to determine if they can be used to estimate discharge.

The predicted water surface elevation accuracy of SWOT measurements is 0.1 m/km^2^ (Rodriguez, 2016). Thus, a change in the water surface elevation of at least 0.1 m/km^2^ is detectable. This accuracy can be compared to water surface elevation changes upstream, at the river mouth, and at the ridge peak to determine if a detectable relationship to discharge exists. In order to distinguish between two discharge values by their water surface elevations, their levels must be distinct by at least 0.1 m/km^2^. The five low‐discharge cases tested here all have water surface elevation levels that are distinct from each other by at least 0.1 m at a location 50 km upstream of the mouth (Figure 5a). In the estuary, the water surface elevation is higher than the elevation in the ocean by 0.1, 0.15, 0.2, 0.22, and 0.24 m for *Fr*_*f*_= 0.1, 0.2, 0.3, 0.4, 0.5, respectively (Figure 5a). These differences are not large enough for SWOT to be able to distinguish between all of the *Fr*_*f*_ values. At the river mouth, the water surface elevation differences between the five low‐discharge cases are ≤ 0.1 m and therefore not detectable by SWOT. During high‐discharge conditions, the water surface elevation differences between the five discharge cases are not distinguishable at the river mouth, but are all distinct by more than 0.1 m, 1 km upstream of the mouth (Figure 7a), which means SWOT may be able to estimate discharge from the water surface elevation level 1 km upstream of the mouth during high‐discharge conditions. Offshore, the ridge heights range from 0.03 to 0.85 m, with the values for *Fr*_*f*_<2 lower than 0.1 m and therefore not measurable by SWOT (Figure 9). The ridge heights of the *Fr*_*f*_>1.2 steep shelf cases are all distinct by more than 0.1 m and therefore distinguishable by SWOT. When the shelf slope is 0.001, only the *Fr*_*f*_=3 case has a ridge height above 0.1 m. The cases with a shelf slope of 0.002 have ridge heights above 0.1 m for *Fr*_*f*_ between two and five, but the differences between the values are less than 0.1 m. These results suggest that a detectable relationship between ridge height and discharge is strongest for high‐discharge cases ( *Fr*_*f*_>2) when the shelf is steep (*α* ≥ 0.005).

**Figure 15:**
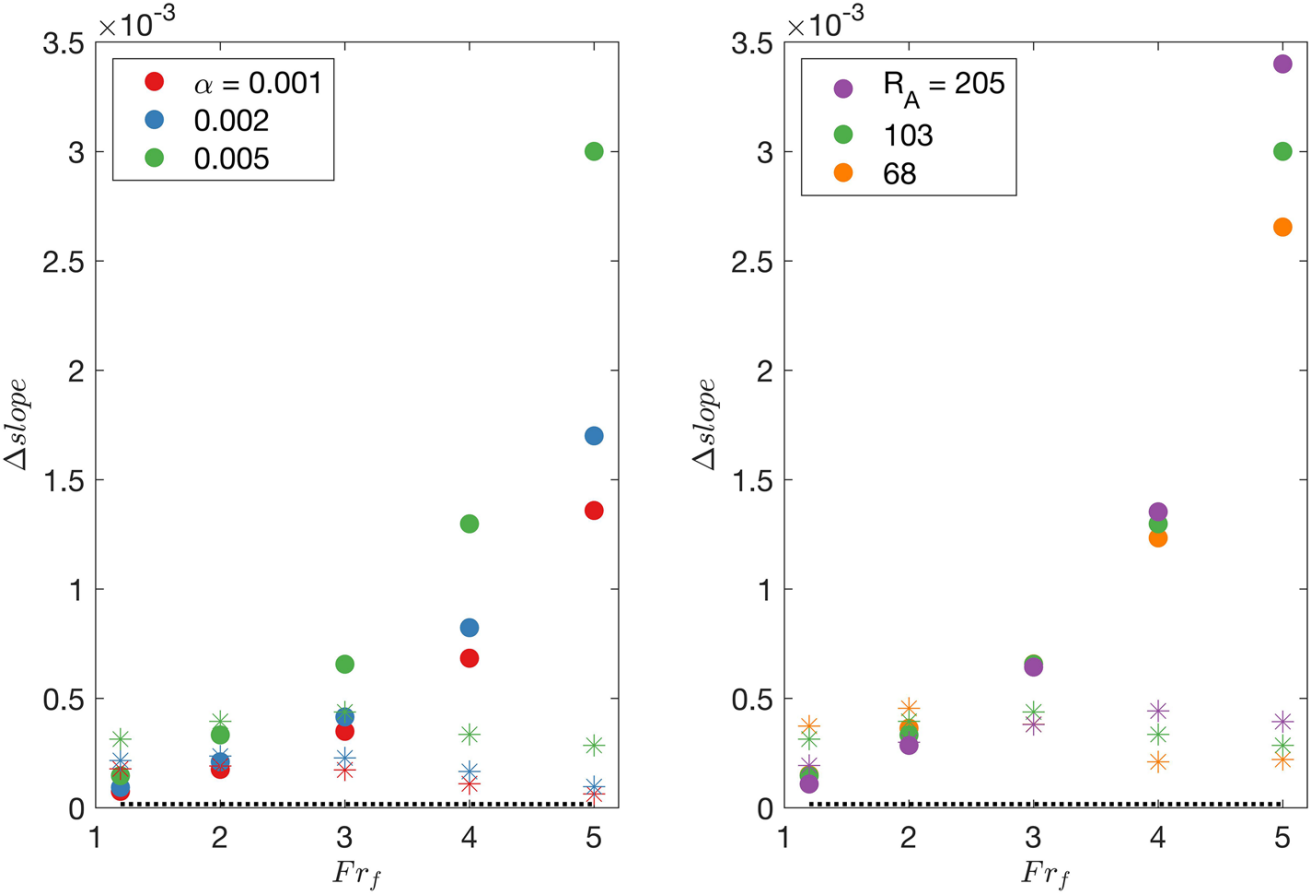
Water surface slope changes at the river mouth (filled circles) and at the ridge peak (stars) for (a) three shelf slopes and (b) three river mouth aspect ratios. The predicted SWOT slope accuracy of 1.7*e*−5 is plotted as a dotted line.

The predicted slope accuracy of the SWOT measurements is 1.7*e* − 5 (Rodriguez, 2016). This can be compared to slope changes at the toe of the salt wedge, the river mouth, and the ridge peak. The slope changes at the toe of the salt wedge were on the order of 6*e* − 6 (Figure 5c), which is below the predicted SWOT accuracy. This implies that SWOT slope measurements will not be able to distinguish between the five low‐discharge runs based on the slope changes at the toe of the salt wedge. The observed slope changes at the mouth during high discharge are on the order of 1.5*e* − 3, which is larger than the predicted slope accuracy of SWOT (Figure 15). The slope changes at the mouth also varied enough between the five high‐discharge cases for SWOT to be able to distinguish between all five for all of the shelf slopes and shoreline depths studied (Figure 15). The slope change at the river mouth also depends on the river bed slope, which makes discharge estimates from that slope change difficult without prior knowledge of river bathymetry. A more robust way to measure discharge remotely from a slope signal would be to locate where liftoff occurs due to the slope changing from positive to negative at the ridge peak. The slope change at the ridge peak is smaller than the slope change at the river mouth (Figure 15), but still larger than the SWOT‐predicted accuracy. It does not increase with discharge, which means it can be used most effectively as a method of determining the liftoff location instead of as a method of directly estimating discharge. Once the liftoff location is determined, then Equation 13 or 15 can be used to calculate discharge.

**Figure 16:**
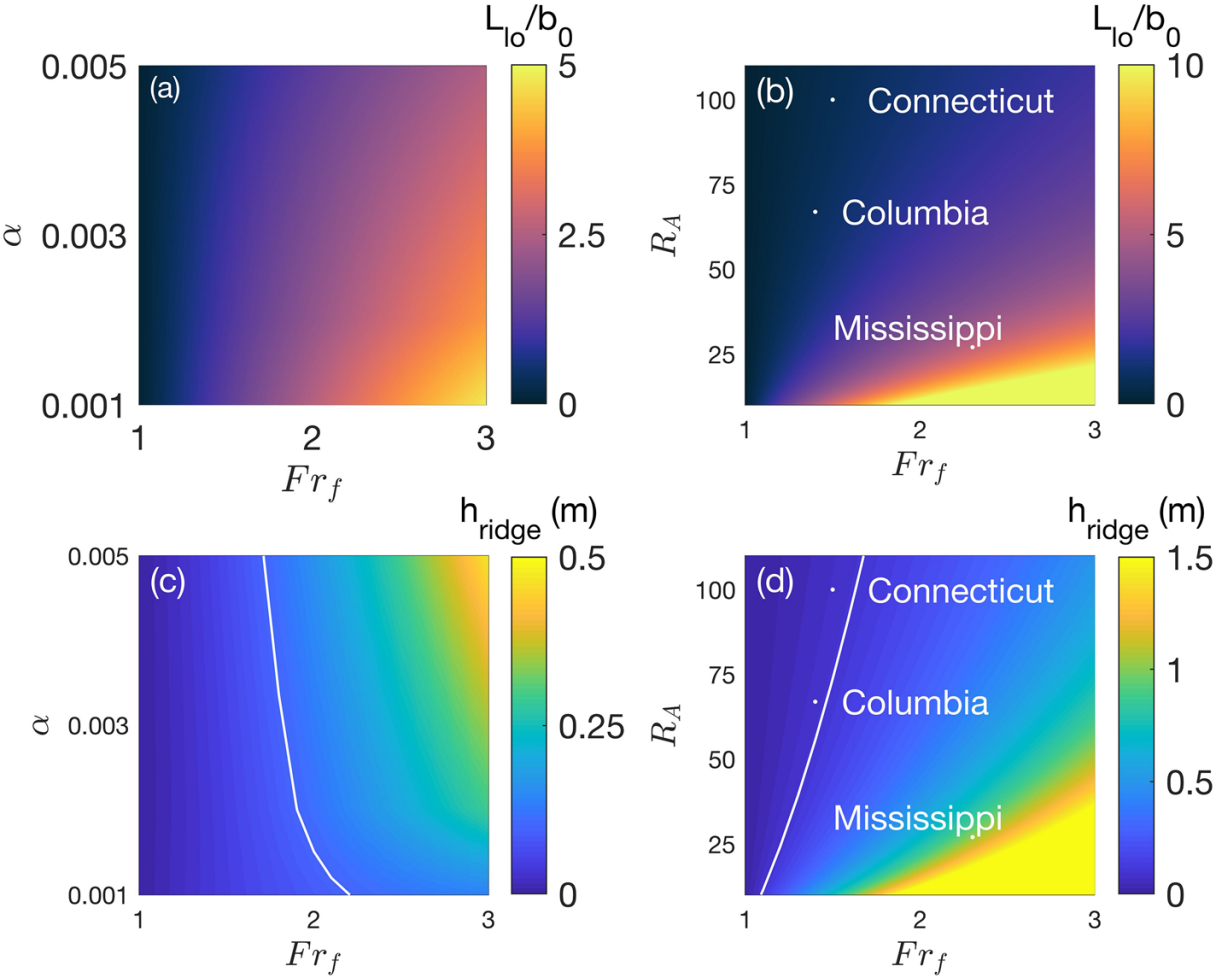
Normalized liftoff lengths and ridge heights for typical aspect ratios and shelf slopes. (a) Normalized liftoff lengths for an aspect ratio of 103 and a range of shelf slopes. (b) Normalized liftoff lengths for a shelf slope of 0.005 and a range of aspect ratios. (c) Ridge heights for an aspect ratio of 103 and a range of shelf slopes with the predicted SWOT accuracy of 0.1 m marked as a white line. (d) Ridge heights for a shelf slope of 0.005 and a range of aspect ratios with the predicted SWOT accuracy of 0.1 m marked as a white line. Possible ridge heights for peak floods of three rivers with different aspect ratios assuming the shelf slope for all of them is 0.005.

The results presented here cover a range of shelf slopes, aspect ratios, and freshwater Froude numbers, which may have water surface signals observable by SWOT. Although rivers such as the Amazon can have aspect ratios greater than 100 and floods with *Fr*_*f*_>5, smaller rivers typically have values of *R*_*A*_ on the order of 10–100 and *Fr*_*f*_ on the order of 1–3. In Figure 16 we investigate the range of liftoff lengths and ridge heights that are predicted by Equations 13 and 18, in terms of *Fr*_*f*_, *α*, and *R*_*A*_. For a fixed aspect ratio of 103, the liftoff length is highest when the shelf slope is shallow and *Fr*_*f*_ is high (Figure 16a). For comparison, predictions for the Connecticut, Columbia, and Mississippi rivers are shown that correspond to typical values of *R*_*A*_ and *Fr*_*f*_ during flood conditions. The derived equations predict liftoff lengths of 500, 1,500, and 10,000 m and ridge heights of 0.07, 0.08, and 1.05 m for the Connecticut, Columbia, and Mississippi Rivers, respectively (Figures 16b and 16d). These predictions suggest that the ridge heights for the Connecticut and Columbia Rivers may be just below the threshold of detection by SWOT, but the Mississippi River may be above it. Therefore, the ridge may be detectable by SWOT in the water surface elevation signal of the Mississippi River but only detectable in the water surface slope signals of the Connecticut and Columbia Rivers. Once the ridge peak is located using the surface slope signals of smaller rivers like the Connecticut and Columbia, then the discharge can be calculated using our derived equation for the discharge in terms of the liftoff length.

## 7 Conclusions

In this study we use a three‐dimensional numerical model to investigate the dependence of the river plume liftoff process on river discharge, shelf slope, and the river mouth aspect ratio. The modeled liftoff location and the water surface elevation at the liftoff location agree well with analytical expressions derived assuming that the upper‐layer Froude number is unity, and the primary order steady state cross‐shelf momentum balance is between advective acceleration, barotropic pressure gradient, and the bottom stress.

We identified water surface elevation signals related to the liftoff process that could be useful for remote sensing of river discharge during high‐ (*Fr*_*f*_>1) and low‐ (*Fr*_*f*_<1) discharge conditions. During high‐discharge conditions a ridge forms at the liftoff point on the water surface. The ridge height varies with discharge, but for most rivers it is too small to be detected by the elevation measurement of the upcoming satellite altimeter SWOT. The surface slope change at the ridge peak is detectable by SWOT's slope measurement, but it does not increase with discharge. A possible method to estimate high flow discharge from a SWOT measurement is to locate the ridge peak by its slope change and then use the equations derived here for liftoff length to calculate discharge. The equations derived here predict liftoff lengths and ridge heights that compare well with the results obtained from the numerical model. Plume spreading is found to be negligible between the river mouth and liftoff for shelf slopes steeper than 0.005, further simplifying the relationship between liftoff location and discharge. The liftoff ridge is still present with the addition of moderate amplitude tides, but the ridge location and height are modulated.

During low‐discharge conditions water surface slope and elevation changes can also be related to discharge; however, their magnitudes are smaller than will likely be detectable by SWOT. This study does not account for changes in water surface elevation due to waves, upwelling, and downwelling, which are expected to further influence the structure of the water surface elevation field near the river mouth. Investigation of these processes is left for future work. Finally, results from this study indicate that low‐ and high‐discharge conditions can be distinguished by the presence or absence of the ridge and SWOT measurements of the location or height of the ridge can be used to estimate discharge. Although these results show some conditions under which SWOT will not be able to measure discharge near the river mouth, we expect future altimeters to have even higher slope and vertical resolution capabilities than SWOT. Future geostationary satellites with high‐resolution altimeters could be positioned above key rivers in remote areas of the Arctic to measure discharge where it is difficult to maintain in situ gauges.

## Data Availability

The numerical code used for this work is open source and located at myroms.org. Model results can be found at this site (https://doi.org/10.5281/zenodo.3468551).

## Appendix A Derivations

### A.1

#### A.1.1 Ridge Height Derivation

(A1) $$ū\frac{dū}{dh_{*}}=-g\frac{d\eta }{dh_{*}}-\frac{C_{D}{ū}^{2}}{h\delta }$$

The dimensionless plume thickness is defined as *h*_∗_=1+*αx*/*h*_*s*_, *C*_*D*_ is the drag coefficient, and *δ*=*α*/*h*_*s*_. Substitute in *Q*/*A* for ū

(A2) $$\frac{Q}{A}\frac{d}{dh_{*}}\frac{Q}{A}=-g\frac{d\eta }{dh_{*}}-\frac{C_{D}}{h\delta }\frac{Q^{2}}{A^{2}}$$

Differentiate *Q*/*A* with respect to *h*_∗_

(A3) $$\frac{-Q^{2}}{A^{3}}\frac{dA}{dh_{*}}=-g\frac{d\eta }{dh_{*}}-\frac{C_{D}}{h\delta }\frac{Q^{2}}{A^{2}}$$

Solve for $\frac{d\eta }{dh_{*}}$

(A4) $$\frac{d\eta }{dh_{*}}=\frac{Q^{2}}{A^{3}g}\frac{dA}{dh_{*}}-\frac{C_{D}}{h\delta g}\frac{Q^{2}}{A^{2}}$$

An equation for *A* can be written using Equations 11 and 12, *h*=*h*_*s*_ *h*_∗_, and Γ=2*κh*_*s*_/(5*αb*_0_)

(A5) $$A=h_{s}h_{*}b_{0}\exp\left(\frac{\Gamma }{Fr_{f}}(h_{*}^{5/2}-1)\right)$$

The variables *h*_*s*_,*b*_0_, and *α* are shown on Figure 1. Differentiate with (A5) respect to *h*_∗_ to obtain

(A6) $$\frac{dA}{dh_{*}}=b_{0}h_{s}\exp\left[\frac{\Gamma }{Fr_{f}}(h_{*}^{5/2}-1)\right]\left[\frac{5\Gamma }{2Fr_{f}}h_{*}^{5/2}+1\right]$$

Substitute (A5) and (A6) into (A4)

(A7) $$\frac{d\eta }{dh_{*}}=\frac{Q^{2}b_{0}h_{s}\exp\left[\frac{\Gamma }{Fr_{f}}(h_{*}^{5/2}-1)\right]\left[\frac{5\Gamma }{2Fr_{f}}h_{*}^{5/2}+1\right]}{gb_{0}^{3}h_{s}^{3}h_{*}^{3}\exp{\left[\frac{\Gamma }{Fr_{f}}(h_{*}^{5/2}-1)\right]}^{3}}-\frac{C_{D}Q^{2}}{h_{*}^{3}\delta gb_{0}^{2}h_{s}^{3}}\left[\frac{1}{\left[\exp{\left[\frac{\Gamma }{Fr_{f}}(h_{*}^{5/2}-1)\right]}^{2}\right)}\right]$$

Simplify to

(A8) $$\frac{d\eta }{dh_{*}}=\frac{Q^{2}}{gb_{0}^{2}h_{s}^{2}}\left[\frac{\frac{5}{2}\frac{\Gamma }{Fr_{f}}h_{*}^{-0.5}+h_{*}^{-3}}{{\left(\exp\left[\frac{\Gamma }{Fr_{f}}(h_{*}^{5/2}-1)\right)\right]}^{2}}\right]-\frac{Q^{2}C_{D}}{h_{*}^{3}\delta gb_{0}^{2}h_{s}^{3}}\left[\frac{1}{{\left[\exp\left[\frac{\Gamma }{Fr_{f}}(h_{*}^{5/2}-1)\right]\right]}^{2}}\right]$$

Integrate to obtain *h*_ridge_

(A9) $$h_{\text{ridge}}=\frac{Q^{2}}{gb_{0}^{2}h_{s}^{2}}{\;\int }_{1}^{1+\frac{\alpha L_{\text{lo}}}{h_{s}}}\frac{\frac{\Gamma }{Fr_{f}}2.5h_{*}^{-0.5}+h_{*}^{-3}}{{\left[\exp\left[\frac{\Gamma }{Fr_{f}}(h_{*}^{5/2}-1)\right]\right]}^{2}}\;dh_{*}-\frac{Q^{2}C_{D}}{\delta gb_{0}^{2}h_{s}^{3}}\int_{1}^{1+\frac{\alpha L_{\text{lo}}}{h_{s}}}\frac{1}{h_{*}^{3}{\left[\exp\left[\frac{\Gamma }{Fr_{f}}(h_{*}^{5/2}-1)\right]\right]}^{2}}dh_{*}$$

Rewrite in terms of *Fr*_*f*_ instead of *Q* and combine terms.

(A10) $$h_{\text{ridge}}=\frac{g^{\prime }Fr_{f}^{2}h_{s}}{g}\;\left\{\int_{1}^{1+\frac{\alpha L_{\text{lo}}}{h_{s}}}\frac{\frac{\Gamma }{Fr_{f}}2.5h_{*}^{-0.5}+h_{*}^{-3}}{\exp\left[\frac{2\Gamma }{Fr_{f}}(h_{*}^{5/2}-1)\right]}\;dh_{*}-\frac{C_{D}}{\alpha }\int_{1}^{1+\frac{\alpha L_{\text{lo}}}{h_{s}}}\frac{1}{h_{*}^{3}\exp\left[\frac{2\Gamma }{Fr_{f}}(h_{*}^{5/2}-1)\right]}dh_{*}\right\}$$

(A11) $$h_{\text{ridge}}=\frac{g^{\prime }Fr_{f}^{2}h_{s}}{g}\;\left\{\int_{1}^{1+\frac{\alpha L_{\text{lo}}}{h_{s}}}\frac{\frac{\Gamma }{Fr_{f}}2.5h_{*}^{5/2}+1-\frac{C_{D}}{\alpha }}{h_{*}^{3}\exp\left[\frac{2\Gamma }{Fr_{f}}(h_{*}^{5/2}-1)\right]}\;dh_{*}\right\}$$

#### A.1.2 Ridge Height Derivation With Negligible Spreading

When spreading is negligible *κ* can be set to 0 and Equation 18 becomes

(A12) $$h_{\text{ridge}}=\frac{g^{\prime }Fr_{f}^{2}h_{s}}{g}\;\left\{\int_{1}^{1+\frac{\alpha L_{\text{lo}}}{h_{s}}}\frac{1-\frac{C_{D}}{\alpha }}{h_{*}^{3}}\;dh_{*}\right\}$$

The depth at liftoff, *h*_lo_, can be substituted in for the upper integration limit.

(A13) $$h_{\text{lo}}=1+\frac{\alpha L_{l0}}{h_{s}}$$

(A14) $$h_{\text{ridge}}=\frac{g^{\prime }Fr_{f}^{2}h_{s}}{g}\;\left\{\int_{1}^{h_{\text{lo}}}\frac{1}{h_{*}^{3}}\;dh_{*}-\frac{C_{D}}{\alpha }{\;\int }_{1}^{h_{\text{lo}}}\frac{1}{h_{*}^{3}}\;dh_{*}\right\}$$

(A15) $$h_{\text{ridge}}=\frac{-g^{\prime }h_{s}}{2g}\;Fr_{f}^{2}\left[(h_{\text{lo}}^{-2}-1)-\left(\frac{C_{D}}{\alpha }(h_{\text{lo}}^{-2}-1)\right)\right]$$

Equation 14 can be used to write *h*_lo_ in terms of *Fr*_*f*_

(A16) $$h_{\text{ridge}}=\frac{-g^{\prime }h_{s}}{2g}\;Fr_{f}^{2}\left[(Fr_{f}^{-4/3}-1)-\left(\frac{C_{D}}{\alpha }(Fr_{f}^{-4/3}-1)\right)\right]$$

which simplifies to

(A17) $$h_{\text{ridge}}=\frac{1}{2}\frac{g^{\prime }}{g}\;h_{s}\left[\left(1-\frac{C_{D}}{\alpha }\right)\left(Fr_{f}^{2}-Fr_{f}^{2/3}\right)\right].$$
